# A modular yeast biosensor for low-cost point-of-care pathogen detection

**DOI:** 10.1126/sciadv.1603221

**Published:** 2017-06-28

**Authors:** Nili Ostrov, Miguel Jimenez, Sonja Billerbeck, James Brisbois, Joseph Matragrano, Alastair Ager, Virginia W. Cornish

**Affiliations:** 1 Department of Chemistry, Columbia University, New York, NY 10027, USA.; 2 Department of Population and Family Health, Mailman School of Public Health, Columbia University, New York, NY 10032, USA.; 3 Institute for Global Health and Development, Queen Margaret University, Edinburgh, UK.; 4 Department of Systems Biology, Columbia University, New York, NY 10032, USA.

**Keywords:** Yeast, biosensor, fungal pathogens, invasive fungal infections, synthetic biology, point-of-care, low-cost diagnostic, G-Protein coupled receptor, lycopene, peptide biomarker

## Abstract

The availability of simple, specific, and inexpensive on-site detection methods is of key importance for deployment of pathogen surveillance networks. We developed a nontechnical and highly specific colorimetric assay for detection of pathogen-derived peptides based on *Saccharomyces cerevisiae*—a genetically tractable model organism and household product. Integrating G protein–coupled receptors with a visible, reagent-free lycopene readout, we demonstrate differential detection of major human, plant, and food fungal pathogens with nanomolar sensitivity. We further optimized a one-step rapid dipstick prototype that can be used in complex samples, including blood, urine, and soil. This modular biosensor can be economically produced at large scale, is not reliant on cold-chain storage, can be detected without additional equipment, and is thus a compelling platform scalable to global surveillance of pathogens.

## Teaser

A yeast-based dipstick biosensor enables low-cost, on-site surveillance of pathogens.

## INTRODUCTION

Global surveillance of pathogens is critical for human health, food security, bioterrorism, and maintenance of biodiversity (*1*, *2*). Although monitoring of global pathogen burden has been traditionally limited to a small number of specialized centers, more effective detection could be performed in real time by making accurate diagnostics accessible at the point of care (*3*). However, these tests are only available for a handful of pathogens and often rely on costly reagents, cold-chain distribution, specialized equipment, and technical personnel (*4*, *5*). In the absence of innovative alternatives to expensive antibody- and nucleic acid–based assays, lengthy culturing methods remain as the dominant approach for monitoring pathogens in resource-limited areas (*3*).

The emerging field of synthetic biology has the potential to provide novel diagnostic platforms to overcome global health challenges (*6*, *7*), much like advances in molecular biology gave rise to antibody diagnostics. Although synthetic biology has thus far been leveraged primarily for economical fermentation of industrial and biomedical commodities via metabolic engineering (*8*), significant applications in public health, animal health, and agriculture remain untapped.

Here, we demonstrate that highly specialized *Saccharomyces cerevisiae* cells can be generated to create transformative on-site diagnostic devices unattainable via traditional engineering methods. Of the growing number of engineered biological systems used for biosensing (*7*, *9*–*11*), *S. cerevisiae* stands out because of its marked robustness, genetic tractability, and established human safety (*12*–*14*). Capitalizing on the orthogonality and specificity of its G protein–coupled receptor (GPCR) mating pathway (*15*), it has also shown potential as a platform for drug discovery by engineering mammalian GPCRs (*16*, *17*).

**Fig. 1:**
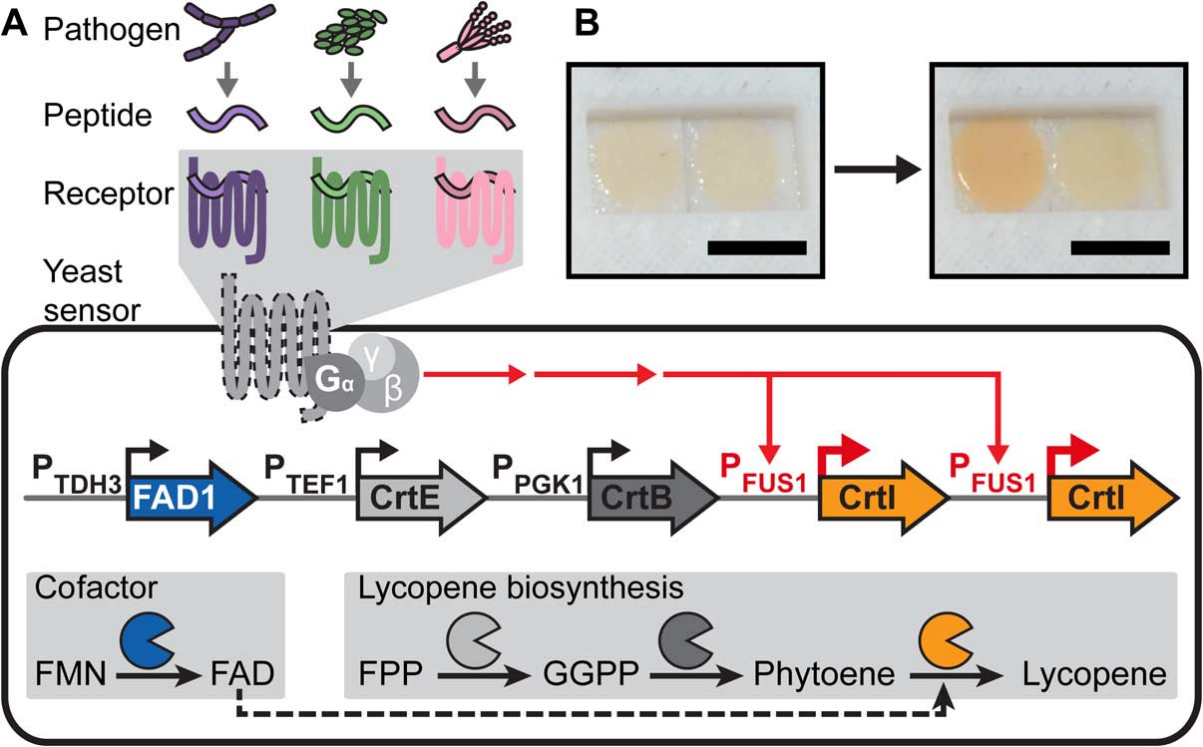
*S. cerevisiae* biosensor for detection of fungal pathogens. (**A**) Overview of biosensor components. Highly specific fungal receptors provide sensitive response to mating peptides secreted by pathogenic fungi. Activation of the downstream mating signaling pathway induces transcriptional activation of biosynthetic genes for production of red lycopene pigment visible to the naked eye. FMN, flavin mononucleotide; FAD, flavin adenine dinucleotide; FPP, farnesyl pyrophosphate; GGPP, geranylgeranyl pyrophosphate. (**B**) Color signal as shown in paper-based dipstick assay. Scale bars, 0.5 cm.

To overcome current bottlenecks in deployment of biosensors, we developed a modular, single-component biosensor that uses fungal mating GPCRs for detection of pathogen-specific peptides and a red plant pigment as readout (Fig. 1). Expanding on well-established methods for scalable and cost-effective distribution of *S. cerevisiae*, we envision that this portable, safe biosensor can enable routine on-site pathogen surveillance.

We demonstrate the utility of our platform for detection of fungal pathogens, a rising global public health burden particularly acute in developing countries (*2*, *18*, *19*). Fungal pathogens cause an estimated 2 million deaths annually and inflict devastating losses of plant crops and population decline in animal wildlife (*2*, *19*). However, efforts to abate fungal infections prevalent in resource-limited areas are hampered by the dearth of fungal diagnostics (*20*). We thus used the large reservoir of natural fungal GPCRs to construct *S. cerevisiae*–based biosensors for detection of fungi responsible for major human disease, agricultural damage, and food spoilage.

## RESULTS

### *S. cerevisiae* as a biosensor visible to the naked eye

We started by building a prototype biosensor for detection of *Candida albicans*, a commensal human pathogen and the leading cause of life-threatening infections in immunocompromised patients (*18*). *C. albicans* is among the most genetically tractable pathogenic fungi, with a well-studied mating system (*21*). For specific detection of the *C. albicans* mating peptide, we replaced the *S. cerevisiae* mating receptor (Sc.Ste2) with that of *C. albicans* (Ca.Ste2) (tables S1 and S2). We then used synthetic *C. albicans* mating pheromone to test receptor activation using a fluorescent reporter that is induced by the mating signaling pathway (*17*).

**Fig. 2:**
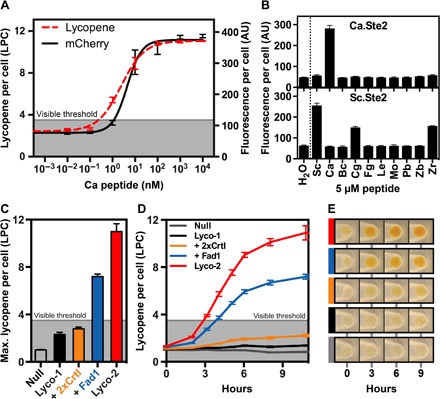
Biosensor functionality and lycopene optimization. (**A**) Activation of *C. albicans* mating receptor (Ca.Ste2) in *S. cerevisiae* by its cognate mating peptide. Fluorescence (black) and lycopene absorbance (red) were used as a transcriptional readout for receptor activation. (**B**) Specificity of Ca.Ste2 and Sc.Ste2 receptors. Fluorescence was determined after 9 hours using 5 μM synthetic fungal peptides. (**C**) Optimization of lycopene production. Maximal lycopene yield was measured after induction with 10 μM synthetic *S. cerevisiae* mating peptide. Null, parental strain (no lycopene genes); Lyco-1, parental strain with single-copy CrtE, CrtB, and CrtI; 2xCrtI, Lyco-1 with additional plasmid-borne copy of CrtI; Fad1, Lyco-1 with additional plasmid-borne copy of Fad1; Lyco-2, all genes genomically integrated into Lyco-1. (**D**) Time course of lycopene production in lycopene-producing strains. Induction as in (C). (**E**) Representative photos of cell pellets (5 × 10^7^ cells) corresponding to strains in (D). Lycopene per cell was determined by spectroscopy (see Supplementary Methods).

Consistent with previous reports (*22*), we found Ca.Ste2 to be highly sensitive to its cognate ligand, with an EC_50_ value (concentration of peptide required for half-maximal activation) of ~4 nM (Fig. 2A). To probe receptor specificity further, we also tested Ca.Ste2 activation using nine heterologous fungal mating peptides and found it to be highly specific to its mating peptide (Fig. 2B).

To meet the needs of on-site detection, we then replaced the fluorescent readout with a robust signal that is easily visible to the naked eye. We chose lycopene, an antioxidant carotenoid pigment naturally produced in plants and bacteria and widely used for metabolic engineering (*23*). We introduced the lycopene production pathway into our strain by placing the first two biosynthetic genes, *Erwinia herbicola crtE* (geranylgeranyl diphosphate synthase) and *crtB* (phytoene synthase), under constitutive promoters from *ADH1* and *TEF1*, respectively. To tie lycopene production to activation of the mating receptor, we placed the last pathway gene *crtI* (lycopene synthase) under control of the pheromone-inducible promoter from *FUS1* (Fig. 1 and fig. S1).

To circumvent the low-throughput chemical extraction that is traditionally used for measurement of lycopene yield (*23*), we developed a quantitative absorbance-based method to measure lycopene production per cell (LPC) (Supplementary Methods). We determined ≥3.5 LPC units to be a stringent threshold for a robust yes/no lycopene readout visible to the naked eye.

Lycopene production in this initial strain reached a maximum of 3 LPC units, just below the visible threshold (Fig. 2, C to E). We thus optimized lycopene production by adding another copy of the endogenous flavin adenine dinucleotide synthase (*FAD1*) (*24*) and lycopene synthase (*crtI*) (fig. S1). The enhanced strain exhibited strong lycopene production of more than 10 LPC units, crossing the visible lycopene threshold in only 3 hours (Fig. 2, D and E).

Using the lycopene readout, we measured the limit of detection (LoD) of our *C. albicans* biosensor in liquid culture to be 1 to 10 nM mating peptide, with lycopene sensitivity closely matching that of the fluorescent reporter (Fig. 2A). The LoD and strong lycopene readout remained stable across pH and temperature and in clinically relevant samples such as urine and serum (fig. S2). Together, these results establish the feasibility of using *S. cerevisiae* as a specific and sensitive detector, competitive with culture-based diagnostic methods.

### Fungal GPCRs as modular detection elements for invasive fungal pathogens

**Fig. 3:**
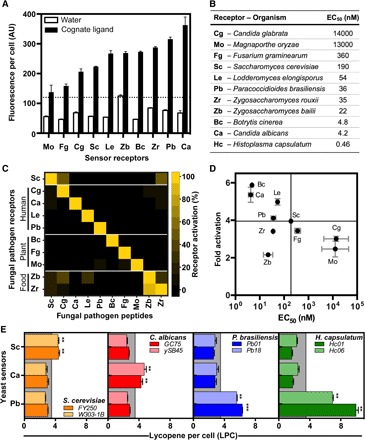
Yeast biosensor for multiple fungal peptides. (**A**) Activation of fungal mating receptors in *S. cerevisiae* by the corresponding cognate synthetic mating peptides (40 μM) (see also fig. S4). Dotted line denotes the effective visible threshold from Fig. 2A. (**B**) EC_50_ values calculated for fungal receptors in *S. cerevisiae* using cognate ligands. (**C**) Specificity of heterologous fungal receptors. Receptors were activated by 5 μM synthetic peptides. Response was measured by fluorescence and normalized for each receptor (see fig. S5). (**D**) Comparative scoring of all biosensors. (**E**) Lycopene production induced by culture supernatant from clinically isolated fungal pathogens. Lycopene per cell measured by spectroscopy at 9 hours (see Supplementary Methods). **P ≤ 0.01, ***P ≤ 0.001; *n* = 3.

Next, we sought to expand the range of target pathogens. However, contrary to *C. albicans*, the mating systems for most fungal pathogens are not well studied (*25*). To identify candidate mating receptors, we searched the large reservoir of available fungal genomes for receptors and putative mating peptides homologous to *S. cerevisiae* *STE2* and *MF*α*1*, respectively (table S1 and fig. S3A) (*26*). We introduced codon-optimized or wild-type receptor genes into an *S. cerevisiae* strain containing a fluorescent reporter (yMJ183) and measured receptor activation using the cognate fungal mating peptides (Fig. 3A, fig. S4, and table S2).

By simply swapping the Ste2 receptor, we generated functional biosensors for 10 major human, agricultural, and food spoilage pathogens: *Candida albicans*, *Candida glabrata*, *Paracoccidioides brasiliensis*, *Histoplasma capsulatum*, *Lodderomyces elongisporus*, *Botrytis cinerea*, *Fusarium graminearum*, *Magnaporthe oryzae*, *Zygosaccharomyces bailii*, and *Zygosaccharomyces rouxii*. Notably, no additional engineering was necessary for activation of these heterologous receptors, as is often required for mammalian GPCRs, possibly due to higher homology with the endogenous receptor (fig. S3).

All receptors exhibited high sensitivity to their cognate ligands, with EC_50_ values ranging from 14 μM to 4 nM (Fig. 3B and fig. S4). Codon optimization of the receptor gene provided a slight increase in sensitivity (fig. S4B).

Most tested receptors were also highly specific to their cognate mating peptide, as expected for mediators of species-specific signals (Fig. 3C and fig. S5). Notably, our results show differential detection of *C. albicans*, *C. glabrata*, *L. elongisporus*, and *P. brasiliensis*, major human pathogens with distinct susceptibility to antifungal drugs (*27*).

We then turned to implementing our lycopene readout for these new fungal targets. Comparison of the fluorescent activation profile of these biosensors with that of our initial *C. albicans* biosensor suggested that the new set of receptors could be readily implemented with the optimized lycopene readout strain to give a strong readout visible to the naked eye (Figs. 2A and 3A). We chose the biosensor for *P. brasiliensis* for further characterization. As with the *C. albicans* biosensor, we observed a *P. brasiliensis* pheromone LoD of <0.01 to 10 nM and a 3.9 ± 0.1 fold activation of lycopene production, yielding a robust readout across pH and temperature and in urine and serum (fig. S6).

### Detection of fungal pathogen clinical isolates

Next, we challenged our biosensor for detection of naturally secreted mating peptides using clinically isolated *Paracoccidioides* strains. Paracoccidioidomycosis (PCM), an invasive fungal infection endemic to Latin America, is one of many neglected tropical diseases that primarily affect poor populations and lack systematic surveillance (*28*). PCM is caused by inhalation of airborne conidia produced by mycelium of the soil ascomycete *P. brasiliensis* (*29*). Recent identification of the genetic components underlying its mating system (*30*) enabled us to pursue specific yeast-based detection of *P. brasiliensis*, which could facilitate detection of its environmental reservoir.

Specifically, we challenged our biosensor to detect cultured mycelial *P. brasiliensis* isolated from human patients (see Materials and Methods). Biosensor cells expressing *P. brasiliensis* mating receptor, which exhibited specific and sensitive detection of its synthetic mating peptide (figs. S5A and S6), were mixed with spent supernatants from two clinically isolated *Paracoccidioides* strains (table S2). In response, we observed lycopene production well above the visible threshold (Fig. 3E). Secreted mating peptides were similarly detected from clinical isolates of *C. albicans* and *H. capsulatum* (Fig. 3E). The peptide produced by *H. capsulatum* (*30*), the causative agent of histoplasmosis (*18*), is identical to that of *P. brasiliensis* and could be detected using both biosensor strains (fig. S7).

### A low-cost, low-tech dipstick assay

**Fig. 4:**
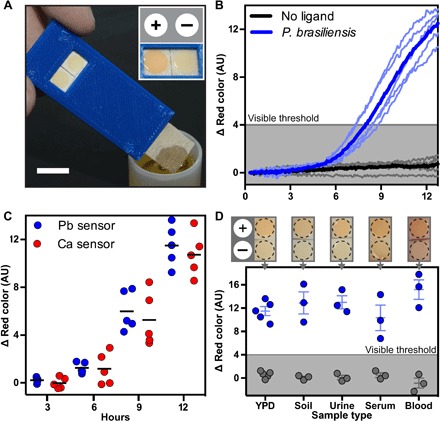
Paper-based dipstick assay for detection of fungal pathogens. (**A**) Dipstick device. Inset: “+,” positive biosensor strain; “−,” negative control strain. (**B**) Quantitative analysis of lycopene production using dipstick assay, as scored by time-lapse photography (see Supplementary Methods) for detection of 1 μM synthetic *P. brasiliensis* mating peptide. Individual runs are shown in light color, and average response is shown in dark color. Shading indicates visible threshold. (**C**) *P. brasiliensis* and *C. albicans* mating peptides were reproducibly detected using the dipstick assay. Maximal response was achieved by 12 hours after exposure to the respective peptides (1 μM). (**D**) Detection of *P. brasiliensis* mating peptide in complex samples. Liquid samples were supplemented with synthetic *P. brasiliensis* mating peptide (blue) or water (gray), and scored as in (B). YPD, medium only; soil, standard potting soil; urine, 50% pooled human urine; serum, 50% human serum; blood, 2% whole blood. All experiments were performed using 1 μM peptide and supplemented with YPD medium. AU, arbitrary units.

Encouraged by these results, we then focused our efforts toward translation of our assay to the field by developing a simple dipstick test. For this aim, biosensor and control cells were spotted onto filter paper, and detection was performed by simply dipping the paper into liquid samples containing synthetic mating peptides (Fig. 4A and fig. S8). In addition to visual inspection, we quantified lycopene accumulation on paper using pixel color analysis (Supplementary Methods).

Using a *P. brasiliensis* dipstick assay, we observed a robust and highly reproducible signal that surpassed the visible lycopene threshold to give a clear yes/no readout (Fig. 4B and movie S1). Similar results were achieved using a *C. albicans* dipstick assay (Fig. 4C and fig. S9). As expected, no cross-reactivity was observed between these two pathogens (movie S1). Last, to ensure that the signal remains visible in complex samples, we performed dipstick tests in soil, urine, serum, and blood supplemented with synthetic mating peptides. In all sample types, micromolar levels of peptide were successfully detected (Fig. 4D, fig. S9, and movies S2 to S5). The dipstick assay retained its functionality after being stored for 38 weeks at room temperature (fig. S10).

## DISCUSSION

In summary, our results show the feasibility and robustness of pathogen detection using engineered *S. cerevisiae*. We achieved sensitivity and specificity levels comparable to those of mammalian, whole-cell, antibody, and nucleic acid assays, all of which are significantly more expensive and technically demanding. Our assay can be constructed cheaply on a large scale by leveraging existing infrastructure for yeast culture, widely distributed as a stable dried product for household use, robustly applied to complex samples, and readily detected by eye.

Although the lycopene readout already provides a robust yes/no response, that response can be further enhanced through promoter optimization, signal amplification feedback loops, alternate lycopene biosynthetic enzymes, or engineering of receptor/G protein interaction. Additionally, although the GPCRs already show marked specificity, total assay specificity can be increased by incorporating additional positive and negative biosensor spots for other target and off-target pheromones or by engineering the pheromone specificity of individual receptors.

Fungal pheromones represent a promising class of species-specific biomarkers that may be used to overcome the time-consuming methods in place today for the definitive diagnosis of fungal pathogens (*3*, *18*). The biosensors developed in this work will simplify the validation of these peptide biomarkers, which remain poorly characterized in clinical and environmental samples (*31*). While at an early stage of implementation, these biosensors can be immediately adopted in the clinic to shorten the time required for diagnosis of fungal pathogens from blood cultures (see Supplementary Methods). Furthermore, pathogen characterization can be expanded to other fungi by adding new GPCR-pheromone pairs, identified through fungal genome mining, to our biosensor platform.

The range of biosensor targets can be further extended beyond fungal pheromones by leveraging established GPCR-directed evolution methods in yeast (*16*, *17*). Fungal GPCRs, as well as other GPCR classes, can be engineered to recognize novel peptide, protein, polysaccharide, and small-molecule ligands as potential targets for yeast-based detection of bacteria, viruses, toxins, and other diseases.

Although early success has established the impact of synthetic biology in the field of metabolic engineering, the application of synthetic biology to grand challenges in biomedicine, materials science, and other fields is still at an early stage of development. Here, we show that yeast engineered with the growing toolbox of synthetic biology can have a major impact on the field of biosensors. While sophisticated, highly technical diagnostics remain limited to clinical and industrial laboratories, reliable low-tech tools accessible to the general population can provide disruptive cheap alternatives to broadly transform the field of diagnostics.

## MATERIALS AND METHODS

### Materials

Synthetic mating peptides (≥95% purity) were obtained from GenScript or Zymo Research. Polymerases, restriction enzymes, and Gibson assembly mix were obtained from New England Biolabs. Culture medium components were obtained from BD Bioscience and Sigma-Aldrich. Primers and synthetic DNA were purchased from Integrated DNA Technologies (IDT). Plasmids were cloned and amplified in either *Escherichia coli* TG1 or C3040 (New England Biolabs). Human urine (catalog no. IR100007P) and single donor human whole blood (catalog no. IPLA-WB1) were purchased from Innovative Research. Human serum, normal off the clot (frozen) (catalog no. HSER-2ML) was purchased from Zen-Bio. Professional potting mix soil was purchased from Fafard.

### *S. cerevisiae* general cloning methods

All strains were derived from parental *reiterative recombination* acceptor strain LW2591 (*MATa-inc* genotype), and cloning of expression modules into the *HO* locus was performed using *reiterative recombination* (*32*). Scarless gene deletions and gene replacements were carried out using *delitto perfetto* (*33*). Endogenous yeast promoters, terminators, and open reading frames (ORFs) were obtained by polymerase chain reaction (PCR) from strain FY251 [American Type Culture Collection (ATCC) 96098] or LW2591 (*32*). Yeast transformations were carried out using the lithium acetate method (*34*). All plasmids are derivatives of the pRS41x series of centromeric shuttle plasmids, and were cloned using standard molecular biology protocols and Gibson assembly (*35*). Yeast strains used in this study are listed in table S2. Plasmids used in this study are listed in table S3. Expression modules constructed in this study are listed in table S4. Primers used for cloning receptors from genomic DNA and plasmid pLPreB are listed in table S5. Codon-optimized and native receptor ORFs used in this study are listed in table S6.

### Determination of fungal GPCRs and mating peptides sequences

Fungal receptors were derived from pathogenic fungi selected from the PFAM (*36*) family PF02116. Sequences were validated by multiple sequence alignment using MUSCLE (*37*), ranging from 21 to 49% identity to wild-type *S. cerevisiae* Ste2. The corresponding peptide ligands were taken from Martin *et al.* (*26*) for species *B. cinerea*, *M. oryzae*, *F. graminearum*, *C. glabrata*, *C. albicans*, *L. elongisporus*, and *Z. rouxii*. The peptide ligand common to species *P. brasiliensis* and *H. capsulatum* was taken from Gomes-Rezende *et al.* (*30*). The peptide ligand for species *Z. bailii* was predicted using the method reported by Martin *et al.* (*26*). The UniProtKB accession numbers for all receptors and sequences of fungal peptides are listed in table S1.

### Cloning of fungal GPCRs

As detailed in table S1, some mating receptor ORFs were synthesized as codon-optimized genes for *S. cerevisiae* (see table S6), and others were cloned directly from the appropriate fungal genomic DNA (ATCC) or plasmid pLPreB using primers in table S5. Codon optimization was performed with the JCat Codon Adaptation tool (*38*) using the default setting for *S. cerevisiae* and further optimized for cloning with the IDT codon optimization tool (Integrated DNA Technologies). All receptor ORFs were incorporated into expression modules containing the *S. cerevisiae TDH3* promoter and *STE2* terminator (see table S4). For fluorescent assays using reporter strain yMJ183, receptor expression modules were cloned into low-copy plasmids derived from pRS416 (table S3). For lycopene biosensor strains, receptor expression modules were integrated at the *STE2* locus.

### Cloning of fluorescent reporter strain

A codon-optimized copy of the mCherry fluorescent reporter gene (*39*) was cloned with the *S. cerevisiae* pheromone-inducible *FUS1* promoter and *ACT1* terminator (table S4). This expression module was integrated at the reiterative recombination acceptor site in strain yMJ105. The *STE2* gene was deleted to yield the fluorescent reporter strain yMJ183. Genotypes of all strains are listed in table S2.

### Cloning of lycopene biosensor strains

The parent lycopene biosensor strain (Lyco-1; yMJ118) was constructed by cloning of lycopene pathway genes from *E. herbicola* at the reiterative recombination acceptor site in strain yMJ105. The *crtE*, *crtB*, and *crtI* ORFs were obtained from plasmid pSC203 (a generous gift from G. Stephanopolous at MIT) (*40*). The *crtE* (geranylgeranyl diphosphate synthase) and *crtB* (phytoene synthase) ORFs were cloned into a constitutive expression module containing promoters from *TEF1* and *PGK1* and the bidirectional terminator from *ADH1*. The *crtI* (lycopene synthase) ORF was cloned into a pheromone-inducible expression module containing the promoter from *FUS1* and the terminator from *ACT1*. The enhanced parent lycopene biosensor strain (Lyco-2; yMJ251) carried an additional copy of the pFUS1-CrtI-tACT1 expression module integrated at the *MET15* locus and a pTDH3-FAD1-tCYC1 overexpression module integrated at the reiterative recombination acceptor site. All fungal biosensor strains described in this study were derived from yMJ251 by replacement of the endogenous *STE2* gene with the appropriate fungal receptor expression module (table S4). Genotypes of all strains are listed in table S2.

### Characterization of GPCR activation in *S. cerevisiae*

Fungal mating receptor Ste2 activity was measured in strain yMJ183 using the fluorescent reporter mCherry. The fluorescent reporter strains carrying the appropriate fungal Ste2 expression plasmid were assayed in 96-well microtiter plates cultured at 30°C and 800 rpm. Cells were seeded at an optical density at 600 nm (OD_600_) of 1 in standard synthetic dropout medium (2% dextrose) lacking the appropriate selective component with either synthetic fungal mating peptide or water (control) as indicated. All measurements were performed in triplicate. mCherry fluorescence (excitation, 588 nm; emission, 620 nm) and culture turbidity (OD_600_) were measured using an Infinite M200 plate reader (Tecan). The OD_600_ value was corrected using eq. S6 (Supplementary Methods). To determine EC_50_ and fold activation values, the fluorescence response of strain yMJ183 carrying the appropriate Ste2 receptor was measured at different concentrations of the appropriate synthetic mating peptide. All raw fluorescence values were normalized by the OD_600_ and plotted against the corresponding peptide concentration, and the points fit with a four-parameter logistic curve using Prism (GraphPad). Fold activation was calculated for each receptor strain as the maximum OD_600_-normalized fluorescence of peptide-treated cells divided by the OD_600_-normalized fluorescence value of water-treated cells. For cross-reactivity measurements, receptor activation was individually measured using each of the synthetic fungal mating peptides (5 μM). Percent receptor activation was calculated by setting the OD_600_-normalized fluorescence value of cognate-peptide activation to 100% and the value of water-treated cells to 0% (see gray regions in fig. S5A).

### Characterization of the lycopene readout in liquid culture

Induction of lycopene was assayed using strain yMJ118 or yMJ251 in 96-well microtiter plates cultured at 30°C and 800 rpm. Cells were seeded at an OD_600_ of 2 in standard complete synthetic medium (2% dextrose) supplemented with 5% yeast extract peptone dextrose (YPD) medium and with the indicated concentration of synthetic peptide. All measurements were performed in triplicate. Cell density and medium conditions were chosen to more closely mimic conditions relevant for the yeast-based paper assay (that is, high cell density and nonselective complex medium) while enabling more precise spectroscopic determination of lycopene content (that is, higher bulk signal at early time points and low-absorbance medium). Under these conditions, the cultures grew to a maximal OD_600_ of 6. Relative lycopene content was calculated by spectroscopy (*41*) using eq. S5 (Supplementary Methods). Optical densities were measured with an Infinite M200 plate reader (Tecan). Lycopene values were normalized by the culture OD_600_ to give a measure of lycopene per cell. For each strain, maximum yield of lycopene per OD_600_ was determined as the largest observed value over a 72-hour period for each biological replicate. Half-maximal lycopene per cell for each biological replicate was calculated as the average of the largest and smallest lycopene per OD_600_ value observed over a 72-hour period. Time to half-maximal lycopene per cell for each strain was determined by linear interpolation between the two time points with lycopene per OD_600_ values that spanned the calculated half-maximal lycopene per OD_600_ for each biological replicate.

### Characterization of biosensor strains in liquid culture (pH, temperature, and complex samples)

*P. brasiliensis* or *C. albicans* biosensor strains (yMJ258 and yMJ260, respectively) were characterized in 96-well microtiter plates cultured at 800 rpm. Cells were seeded at an OD_600_ of 2 in standard complete synthetic medium (2% dextrose) supplemented with 5% YPD medium and the indicated concentration of synthetic peptide. A 2× stock of medium and a 10× stock of the ligand were diluted to reach the appropriate 1× concentration. All measurements were performed in triplicate (figs. S2 and S6). Lycopene production was measured by absorbance as described above. For temperature assays, the microtiter plate shaker was preequalized to the appropriate temperature for 1 hour before the start of the assay. For pH assays, the pH of the medium stock was titrated to the appropriate value with sodium hydroxide. For complex sample assays, urine and serum were centrifuged to remove particulates and used at a final concentration of 50% (in 1× YPD). The LoD was defined as the lowest experimentally determined peptide concentration giving a lycopene signal significantly above the water-treated sample (*P* ≤ 0.01). *P* values were determined by performing a paired parametric *t* test comparing peptide- and water-treated samples in Prism (GraphPad).

### Preparation of culture supernatant from clinically isolated fungal pathogens

#### H. capsulatum

Supernatants from strains Hc01 and Hc06 were a gift from C. Rappleye (Department of Microbiology, Ohio State University, Columbus, OH). These strains are clinical isolates representing North America class 2 (NAm2) and North America class 1 (NAm1), respectively (*42*). *H. capsulatum* strains were added to liquid sabouraud dextrose agar medium [glucose (40 g/liter) and peptone (10 g/liter)] at 10^5^ cells/ml and incubated for 10 days at 26°C without agitation to induce conversion to mycelia. Conversion to mycelia was confirmed by phase-contrast microscopy. Mycelia were then transferred to *Histoplasma* macrophage medium (*43*), and the cultures were incubated at 26°C. After 3 weeks of growth, mycelia were separated from the supernatant by filtration through a cellulose filter (Whatman qualitative filter paper #2, 8-μm-diameter pores) and the filtrate was subsequently filtered through a polyethersulfone membrane (0.45-μm-diameter pores) to obtain the final culture filtrate. The supernatants were lyophilized, resuspended in 0.1 volume of H_2_O (10× concentration), and kept at −20°C.

#### *Paracoccidioides* spp.

Supernatants from *P. brasiliensis* Pb18 and *Paracoccidioides lutzii* Pb01 cultures were a gift from F. Rodrigues [Life and Health Sciences Research Institute (ICVS), University of Minho, Braga, Portugal]. These strains are clinical isolates containing mating loci MAT1-2 and MAT1-1, respectively (*44*). The mycelium form was grown at 24°C at 150 rpm in synthetic McVeigh-Morton liquid medium (*45*). Supernatants were collected by filtration 10 days after the yeast-mycelium transition. The supernatants were lyophilized, resuspended in 0.1 volume of H_2_O (10× concentration), and kept at −20°C.

#### C. albicans

Human isolate GC75 with MTLα/MTLα (*46*) was a gift from R. Bennett (Department of Molecular Microbiology and Immunology, Brown University, Providence, RI). A second human isolate of *C. albicans* was provided by A.-C. Uhlemann (Department of Medicine, Division of Infectious Diseases, Columbia University, New York, NY). This isolate, referred to as ySB36, was genotyped by PCR for its mating loci using genomic DNA as template as previously described (*47*) and found to be heterozygous for its mating loci, MTLa/MTLα (for primers, see table S5). Homozygous MTLα/MTLα derivatives of ySB36 were obtained by selection on sorbose, as previously described (*48*). In brief, ySB36 was cultured for 16 hours in YPD liquid medium at 30°C and washed once with water, and ~10^5^ cells were plated on 2% sorbose medium (0.67% yeast nitrogen base without amino acids, 2% sorbose). Colonies were visible after 4 days of incubation at 30°C. Several colonies were restreaked on 2% sorbose medium, followed by restreaking on YPD medium and genotyping by colony PCR as above. One homozygous MTLα/MTLα isolate (ySB45) was used for supernatant preparation. Phenotypically switched opaque colonies of GC75 and ySB45 were isolated by phloxine B staining, as previously described (*49*). In brief, a single colony of GC75 or ySB45 was incubated for 24 hours at 25°C in liquid YPD medium without agitation. In total, ~2 × 10^3^ cells were plated on YPD agar supplemented with phloxine B (5 μg/ml; Sigma-Aldrich) and incubated at 25°C for 4 days. Opaque colonies stained pink on phloxine B–containing medium. For supernatant preparation, a single opaque colony of *C. albicans* GC75 or ySB45 was cultured overnight in YPD medium at 25°C and used to inoculate 50 ml of YPD liquid medium. Cells were cultured for ~24 hours at 25°C to a final OD_600_ of 9.5 (~2.8 × 10^8^ cells/ml) and 7.9 (~2.3 × 10^8^ cells/ml). Cells were pelleted by centrifugation, and the supernatant was reduced to dryness by vacuum concentration, resuspended in 0.1 volume H_2_O (10× concentration), and kept at −20°C.

#### S. cerevisiae

Samples were obtained from *S. cerevisiae* strain FY250 with MTLα (*50*) and W303-1B with MTLα (ATCC 201238). Cells were cultured in 50 ml of YPD medium for 20 hours at 30°C to a final OD_600_ of 9.8 (~2.9 × 10^8^ cells/ml) and 8.5 (~2.5 × 10^8^ cells/ml), respectively. Cells were pelleted by centrifugation, and the supernatant of FY250 was reduced to dryness by vacuum concentration, resuspended in 0.1 volume H_2_O (10× concentration), and kept at −20°C. The supernatant of W303-1B was kept at 1× concentration at −20°C.

### Detection of mating peptides in supernatants of clinically isolated fungal strains

*P. brasiliensis* or *C. albicans* biosensor strains (yMJ258 and yMJ260, respectively) and a control *S. cerevisiae* strain (yMJ251) were used to test for the presence of the respective mating peptides in supernatants derived from clinically isolated pathogenic fungi or *S. cerevisiae* (supernatant preparation described above). Cells were seeded at an OD_600_ of 2 in the indicated supernatant mixed with standard complete synthetic medium (2% dextrose) supplemented with 5% YPD in 96-well microtiter plates and cultured at 30°C and 800 rpm, and lycopene production was measured by absorbance as described above. A 2× stock of medium and a 10× stock of the supernatant were used and diluted to reach the appropriate 1× concentration. The control supernatant for W303-1B was diluted to 50% in the final assay. Statistical significance of signal (that is, biosensor strain treated with its cognate supernatant) over noise (same biosensor strain treated with noncognate supernatants) was determined by performing a paired parametric *t* test in Prism (GraphPad). The highest *P* value resulting from sample comparisons is given as **P ≤ 0.01 and ***P ≤ 0.001 (Fig. 3E). All measurements were performed in triplicate.

### Paper-based dipstick assay for detection of fungal peptides in complex samples

A simple, low-cost dipstick assay was designed with the biosensor strains spotted on paper as the only required active component (fig. S8). To assemble the dipstick, the biosensor strains were precultured in 50 ml of YPD medium at 30°C at 300 rpm for 72 hours. The culture was diluted with water to an OD_600_ of 2.5 and vacuum-filtered onto a glass fiber filter paper (Thermo Scientific, DS0281-7500) using a plastic stencil to generate spots with a diameter of 5 mm. An appropriate culture volume was used to give about 5 × 10^7^ cells per spot. The filter paper with biosensor spots was cut into small squares (8 mm × 8 mm, one biosensor spot) and placed onto a strip of wicking paper made of a standard brown paper towel (fig. S8, B and C). Each paper-based dipstick assay contained two different spots, an indicator (biosensor) spot and a control spot composed of *S. cerevisiae* carrying an off-target receptor as a negative control (fig. S8B).

To characterize its functionality, the dipstick was dipped into 1 ml of liquid sample and incubated at 30°C. The lycopene readout was visually inspected and quantitatively measured using time-lapse photography analyzed with ImageJ (see Supplementary Methods) (*51*). A 24-well plate was used to easily array several dipsticks in the field of view of the camera. For all assays, a 10× stock of medium was used and diluted to reach the appropriate 1× concentration. All measurements were performed in three or more replicates. For YPD assays (Fig. 4, B to D, fig. S9, and movie S1), the dipstick was dipped into 1× YPD medium supplemented with 1 μM of the indicated fungal pathogen peptide. For soil assays (Fig. 4D, fig. S9B, and movie S2), 0.5 g of soil was preconditioned with 2 nmol (in 200 μl of water) of the indicated fungal pathogen peptide and allowed to air-dry for 1 hour. The dipstick was inserted into the soil and 2 ml of 1× YPD medium was added to give a concentration of 1 μM fungal peptide. For urine and serum assays (Fig. 4D, fig. S9B, and movies S3 and S4), the samples were vortexed briefly to resuspend particles and supplemented with 1× YPD medium to give a concentration of 50% of urine or serum. For blood assays (Fig. 4D, fig. S9B, and movie S5), the sample was supplemented with 1× YPD medium to give a final concentration of 2% blood.

Additionally, we designed a small plastic holder to facilitate the ease of use of this dipstick assay (fig. S8D). This plastic holder was three-dimensionally (3D) printed out of acrylonitrile butadiene styrene. We validated that the holder did not negatively affect the assay functionality (fig. S8E).

### Long-term stability of the paper-based dipstick

To assay the long-term stability of the paper dipstick, the biosensor spots were prepared on filter paper as described above and allowed to air-dry for 20 min at room temperature. The filter papers were then placed in plastic pouches, flushed with argon, sealed, and stored in the dark at room temperature. After 38 weeks of storage, the filter papers were removed from the storage pouches and assembled with the paper towel wicking paper as described above. To characterize their functionality, the assembled paper dipsticks were rehydrated by dipping them directly into 1 ml of liquid sample composed of 1× YPD medium supplemented with either 1 μM of the indicated fungal pathogen peptide or water as a control and incubated at 30°C. The lycopene readout was visually inspected and quantitatively measured using time-lapse photography (see Supplementary Methods). All measurements were performed in three or more replicates.

### Visibility threshold of lycopene readout

When measured spectroscopically (see Supplementary Methods), we determined a visible threshold of 3.5 LPC in liquid cultures. This threshold was determined by visually inspecting pellets of 5 × 10^7^ cells as shown in Fig. 2E. This visible threshold is noted by the gray line in Figs. 2 (A, C, and D) and 3E and figs. S1, S2, and S6. We also determined the corresponding effective visibility threshold for receptors characterized via fluorescent reporters to be 120 fluorescent units per cell, by comparing the lycopene and fluorescent readouts as shown in dose-response curves for the *C. albicans* biosensor (Fig. 2A). This effective visibility threshold is shown by the dotted line in Fig. 3A.

We also determined a visibility threshold for paper-based dipstick assay when measured by time-lapse photography and pixel color analysis (see Supplementary Methods). This was done by visually inspecting time-lapse clips as shown in movie S1. The visible threshold for the dipstick assay was determined to be 4 Δ red color units and is shown by a gray line in Fig. 4 (B and D) and figs. S8E and S9.

## Supplementary Material

http://advances.sciencemag.org/cgi/content/full/3/6/e1603221/DC1

## SUPPLEMENTARY MATERIALS

Supplementary material for this article is available at http://advances.sciencemag.org/cgi/content/full/3/6/e1603221/DC1 Supplementary Methods fig. S1. Optimization of peptide-induced lycopene production. fig. S2. *C. albicans* biosensor robustness in liquid culture. fig. S3. Sequence analysis of fungal mating receptors. fig. S4. Dose-response curves for fungal mating receptors. fig. S5. Specificity of fungal mating receptors. fig. S6. *P. brasiliensis* biosensor characterization in liquid culture. fig. S7. Comparison of mating receptors from human pathogens *P. brasiliensis* and *H. capsulatum*. fig. S8. Paper-based dipstick assay. fig. S9. Detection of *C. albicans* using dipstick assay. fig. S10. Long-term stability of paper-based dipsticks stored at room temperature. table S1. Fungal pathogen peptides and receptor genes used in this study. table S2. Strains used in this study. table S3. Plasmids used in this study. table S4. List of expression modules constructed in this study. table S5. Primers for cloning of fungal receptors and for genotyping of *C. albicans* isolates. table S6. DNA sequence for fungal receptor ORFs used in this study. movie S1. Yeast dipstick assay with plastic holder. movie S2. Yeast dipstick assay in soil. movie S3. Yeast dipstick assay in urine. movie S4. Yeast dipstick assay in serum. movie S5. Yeast dipstick assay in blood. References (*52*–*55*)
