# Supplementary material for: A modular yeast biosensor for low-cost point-of-care pathogen detection
Source: Sci Adv. 2017 Jun 28;3(6):e1603221. doi: 10.1126/sciadv.1603221 (PMC5489263; doi:10.1126/sciadv.1603221)
Supplement: http://advances.sciencemag.org/cgi/content/full/3/6/e1603221/DC1 [file supp_3_6_e1603221__index.html]

Science Advances | Science Advances

## Supplementary Materials

**This PDF file includes:**

- Supplementary Methods
- fig. S1. Optimization of peptide-induced lycopene production.
- fig. S2. *C. albicans* biosensor robustness in liquid culture.
- fig. S3. Sequence analysis of fungal mating receptors.
- fig. S4. Dose-response curves for fungal mating receptors.
- fig. S5. Specificity of fungal mating receptors.
- fig. S6. *P. brasiliensis* biosensor characterization in liquid culture.
- fig. S7. Comparison of mating receptors from human pathogens *P. brasiliensis* and *H. capsulatum*.
- fig. S8. Paper-based dipstick assay.
- fig. S9. Detection of *C. albicans* using dipstick assay.
- fig. S10. Long-term stability of paper-based dipsticks stored at room temperature.
- table S1. Fungal pathogen peptides and receptor genes used in this study.
- table S2. Strains used in this study.
- table S3. Plasmids used in this study.
- table S4. List of expression modules constructed in this study.
- table S5. Primers for cloning of fungal receptors and for genotyping of *C. albicans* isolates.
- table S6. DNA sequence for fungal receptor ORFs used in this study.
- Legends for movies S1 to S5
- References (*52–55*)

Download PDF

**Other Supplementary Material for this manuscript includes the following:**

- movie S1 (.mp4 format). Yeast dipstick assay with plastic holder.
- movie S2 (.mp4 format). Yeast dipstick assay in soil.
- movie S3 (.mp4 format). Yeast dipstick assay in urine.
- movie S4 (.mp4 format). Yeast dipstick assay in serum.
- movie S5 (.mp4 format). Yeast dipstick assay in blood.

Download Movies S1 to S5

**Files in this Data Supplement:**

- Adobe PDF - 1603221\_SM.pdf
