# Supplementary material for: A modular yeast biosensor for low-cost point-of-care pathogen detection
Source: Sci Adv. 2017 Jun 28;3(6):e1603221. doi: 10.1126/sciadv.1603221 (PMC5489263; doi:10.1126/sciadv.1603221)
Supplement: http://advances.sciencemag.org/cgi/content/full/3/6/e1603221/DC1 [file 1603221_SM.pdf]

Science Advances

AAAS

advances.sciencemag.org/cgi/content/full/3/6/e1603221/DC1

Supplementary Materials for

A modular yeast biosensor for low-cost point-of-care pathogen detection

Nili Ostrov, Miguel Jimenez, Sonja Billerbeck, James Brisbois, Joseph Matragrano,
Alastair Ager, Virginia W. Cornish

Published 28 June 2017, *Sci. Adv.* **3**, e1603221 (2017)
DOI: 10.1126/sciadv.1603221

The PDF file includes:

- Supplementary Methods
- fig. S1. Optimization of peptide-induced lycopene production.
- fig. S2. *C. albicans* biosensor robustness in liquid culture.
- fig. S3. Sequence analysis of fungal mating receptors.
- fig. S4. Dose-response curves for fungal mating receptors.
- fig. S5. Specificity of fungal mating receptors.
- fig. S6. *P. brasiliensis* biosensor characterization in liquid culture.
- fig. S7. Comparison of mating receptors from human pathogens *P. brasiliensis* and *H. capsulatum*.
- fig. S8. Paper-based dipstick assay.
- fig. S9. Detection of *C. albicans* using dipstick assay.
- fig. S10. Long-term stability of paper-based dipsticks stored at room temperature.
- table S1. Fungal pathogen peptides and receptor genes used in this study.
- table S2. Strains used in this study.
- table S3. Plasmids used in this study.
- table S4. List of expression modules constructed in this study.
- table S5. Primers for cloning of fungal receptors and for genotyping of *C. albicans* isolates.
- table S6. DNA sequence for fungal receptor ORFs used in this study.
- Legends for movies S1 to S5
- References (*52–55*)

Other Supplementary Material for this manuscript includes the following:
(available at advances.sciencemag.org/cgi/content/full/3/6/e1603221/DC1)

- movie S1 (.mp4 format). Yeast dipstick assay with plastic holder.
- movie S2 (.mp4 format). Yeast dipstick assay in soil.
- movie S3 (.mp4 format). Yeast dipstick assay in urine.
- movie S4 (.mp4 format). Yeast dipstick assay in serum.
- movie S5 (.mp4 format). Yeast dipstick assay in blood.

Supplementary Methods

Determination of lycopene content in microtiter plate format. To determine the relative lycopene content directly in a cell suspension, we adapted the method proposed by Myers et al. (*41*) to characterize pigmented cells through optical density measurements at multiple wavelengths. This method greatly reduces the noise due to variations in cell growth phase, cell density and other sample irregularities. This enabled the precise evaluation of lycopene content in a high throughput microtiter plate format.

As described by Myers et al. (*41*), the optical density of the cell suspension measured at a sensitive wavelength (i.e. corresponding to an absorption maxima of the pigment) is approximately composed of two additive components: scatter due to cells and absorbance due to the pigment. Therefore the pigment content in a cell suspension is proportional to the measured optical density corrected for the scattering component as follows

$$\left[pigment\right] \mu \; Abs_{S,P} = OD_S - OD_{S,scat} \qquad \text{(S1)}$$

where $Abs_{S,P}$ is the absorbance due to the pigment at the sensitive wavelength S, OD_S is the measured optical density at the sensitive wavelength *S*, and $OD_{S,scat}$ is a calculated scattering component at the sensitive wavelength *S*. Since there was noticeable Raleigh-like wavelength dependence in the scatter of lycopene null strains we chose the following functional form to approximate scatter at a particular wavelength λ

$$OD_{l,scat} = B - \log_{10}\left(1 - \frac{A}{l}\right) \qquad \text{(S2)}$$

where *A* and *B* are constants that reflect changes in cell density and other sample irregularities. At each time point and for each sample, we can calculate the corresponding values of *A* and *B* by using the optical density values measured at two robust wavelengths (i.e. corresponding to wavelengths where scatter is the only or dominant component). Substituting these additional scatter-only optical density measurements into Eq. S2 and solving for *A* and *B* we get

$$A = R1\left(\frac{1 - T}{\frac{R1}{R2} - T}\right), \text{ where } T = 10^{OD_{R1} - OD_{R2}} \qquad \text{(S3)}$$

$$B = OD_{R1} + \log_{10}\left(1 - \frac{A}{R2}\right) \qquad \text{(S4)}$$

where OD_{R1} and OD_{R2} are the measured optical densities at the robust wavelengths *R1* and *R2*. Therefore, by setting $\lambda = S$ and substituting Eq. S2 into Eq. S1, the relative content of lycopene in a cell suspension is given by

$$\left[pigment\right] \mu \; Abs_{S,P} = OD_S + \log_{10}\left(1 - \tfrac{A}{S}\right) - B \qquad \text{(S5)}$$

To apply this method to lycopene in yeast, we determined the appropriate sensitive and robust wavelengths by obtaining the absorbance spectrum of lycopene directly in yeast cells. The spectrum was determined by subtracting the optical density spectrum of a lycopene null strain yMJ105 from that of a constitutive lycopene producing strain LW2671 (fig. S1B). This spectrum showed the characteristic profile of lycopene absorbance and had two major absorbance maxima

at 485 nm and 520 nm (fig. S1C). Based on this spectrum, 520 nm was chosen as the sensitive wavelength (S = 520) since it is furthest away from other natural chromophores in yeast that absorb below 500 nm (e.g. flavins). 395 nm and 600 nm were chosen as the two robust wavelengths ($R1$ = 600 and $R2$ = 395) with low absorbance from lycopene and other natural chromophores.

Three additional considerations were crucial to yield reproducible lycopene measurements in a microtiter plate format. First, all three optical density measurements (at 395 nm, 520 nm and 600 nm) were taken at the same time for each well to reduce errors due to the settling of cells during the measurement of a whole microtiter plate. Second, assay wells were blanked using a reference well on the same microtiter plate containing identical media conditions as the assay wells but with no cells. This was particularly important when colored media was used. Finally, high cell densities ($OD_{600} \geq 2$) were used to yield larger bulk lycopene signals even with the short path length of micro titer plates (~3 mm). Since these high optical density values were outside the linear range of the photodetector, all optical density values were first corrected using the following formula to give true optical density values

$$OD_{true} = \frac{k \times OD_{meas}}{OD_{sat} - OD_{meas}} \quad \text{(S6)}$$

where OD_{meas} is the measured optical density, OD_{sat} is the saturation value of the photodetector and k is the true optical density at which the detector reaches half saturation of the measured optical density. Appropriate values for OD_{sat} and k were determined by plotting direct optical density measurements of a range of cultures of several strains, against the true optical densities determined by dilution to the linear range. Optical densities were taken at 395 nm, 520 nm and 600 nm. All points were fit once with Eq. S6 using Prism (GraphPad) to give OD_{sat} = 3.57 and k = 3.16. These values were used to correct all optical density measurements in this study.

Determination of lycopene content by time-lapse photography. To enable quantitative characterization of the paper-based dipstick assay we developed a method to measure lycopene production based on time-lapse photography and pixel color value analysis. Specifically, dipsticks dipped in samples and a tripod-mounted digital single-lens reflex camera (DSLR, Nikon D7000) were placed in a dark box kept at 30 °C. Flash photographs were taken automatically every 5 minutes. The resulting sequence of photographs was analyzed using ImageJ (*51*). For each time point, the average pixel color values were measured for each of the two dipstick spots using constant measurement areas. The apparent level of red color of each spot was first calculated by the following

$$R_{apparent} = \frac{R - \left(\frac{G+B}{2}\right)}{R} \quad \text{(S7)}$$

where R, G, B are the measured red, green and blue color values, respectively. Since the color of the biosensor spots ranges from off-white to red-orange the color values are such that R>G>B is always true. Therefore, $R_{apparent}$ is a value that scores the level of red from 0 to 1. We then calculated the total level of positive lycopene readout produced by the dipstick by the following

$$\Delta Red\ Color = R_{app,\ indicator} - R_{app,\ negative} \quad \text{(S8)}$$

where $R_{app, indicator}$ and $R_{app, negative}$ are the apparent red color values of the indicator biosensor spot and the negative control yeast spot, respectively given by Eq. S7. Importantly, since the two yeast spots of the dipstick assay are always in close proximity to each other, the Δ Red Color value is not sensitive to variations in light levels and can be used to compare dipsticks placed anywhere in the field of view of the camera.

Using these sequences of photographs we also generated time-lapse clips (movies S1 to S5) showing that the lycopene color change can be visualized by the naked eye. These clips are motion and exposure equalized to remove flicker between frames.

Note: We envision the current version of our sensor may be immediately applicable for fungal diagnosis by shortening the time it takes to discriminate fungal contaminant in blood-culture procedure from days to hours. Specifically, current diagnostic of systemic fungal infections rely on a 5-day automated system in which a blood sample is inoculated into a standardized rich media (blood culture) optimized to amplify all potentially present microorganisms (~24h). A positive blood culture is plated on selective media to further diagnose the organisms (~3-4 days) (personal communication with Dr. Anne-Catrin Uhlemann, Columbia University Medical Center). Using our biosensor, the 3-4 day plate culture could be replaced with just several hour direct diagnosis of the blood culture.

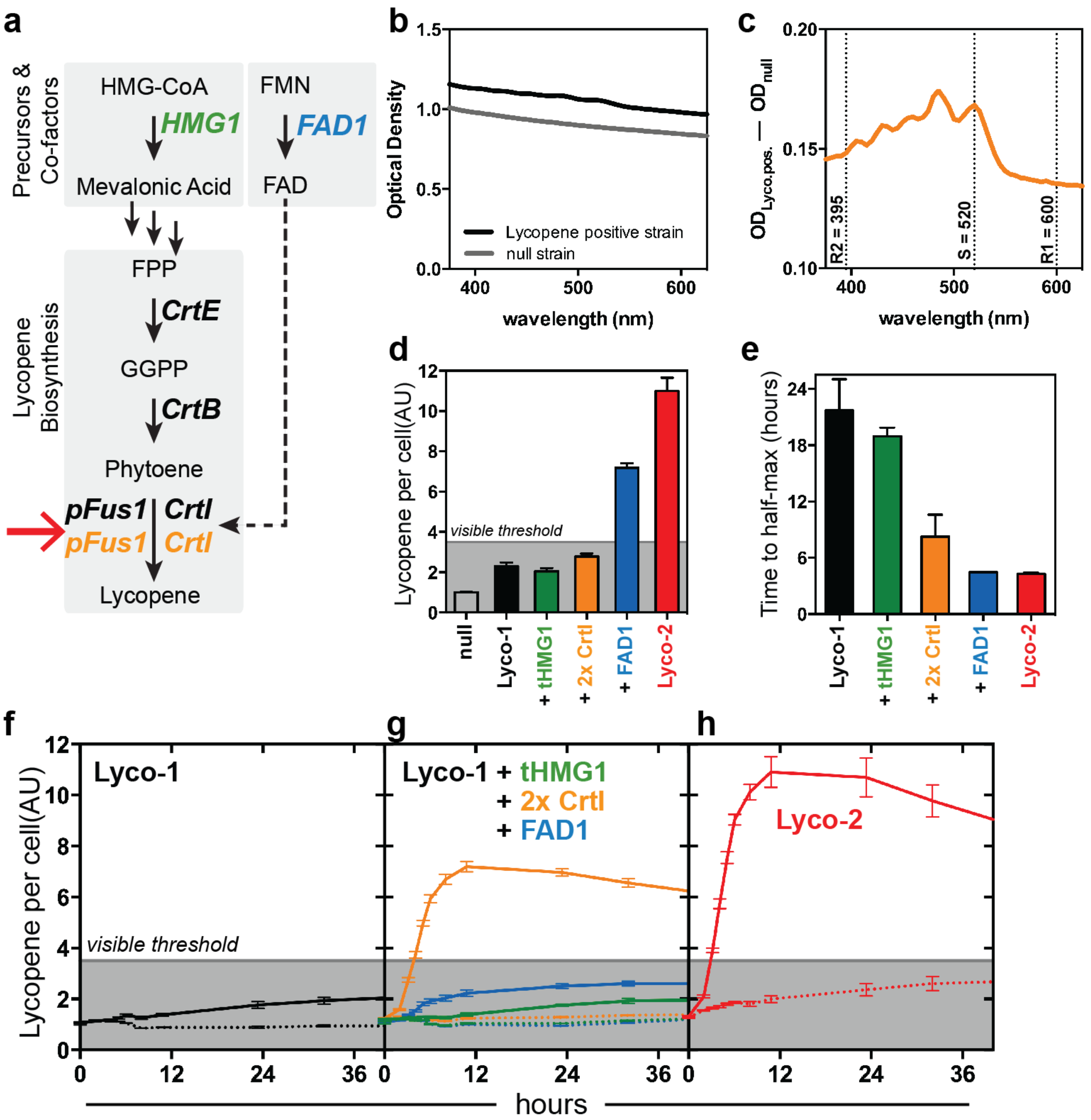

fig. S1. Optimization of peptide-induced lycopene production. (**A**) Lycopene biosynthetic pathway. Lycopene production is induced (red arrow) by mating-signal dependent activation of the *FUS1* promoter. Biosynthetic enzymes shown in bold. Genes targeted for optimization shown in colors. HMG-CoA: 3-hydroxy-3-methylglutaryl-coenzyme A, FMN: flavin mononucleotide, FAD: flavin adenine dinucleotide, FPP: farnesyl pyrophosphate, GGPP: geranylgeranyl pyrophosphate. (**B**) Optical density spectrum of constitutive lycopene producing and lycopene null strains. (**C**) The spectrum of lycopene in yeast cells calculated from B. This spectrum allows selection of wavelengths for spectroscopic measurement of lycopene per cell (see Supplementary Methods). (**D**) Maximal lycopene yield per cell calculated from time course data in F-H. "Null" (grey) - parental strain (no lycopene genes); "Lyco-1" (black) - parental strain with single copy CrtE, CrtB and CrtI; "tHMG1" (green) - Lyco-1 with plasmid-borne truncated copy of Hmg1; "2xCrtI" (orange) - Lyco-1 with plasmid-borne copy of CrtI; "Fad1" (blue) - Lyco-1 with plasmid-borne copy of Fad1; "Lyco-2" (red) - Lyco-1 with additional genes genomically integrated. (**E**) The time to half-maximal lycopene yield was used to compare readout speed. Strains as in D. (**F** to **H**), Time course of lycopene strains induced with 10 µM of *S. cerevisiae* peptide (solid line) or water (dotted line). Strains as in D.

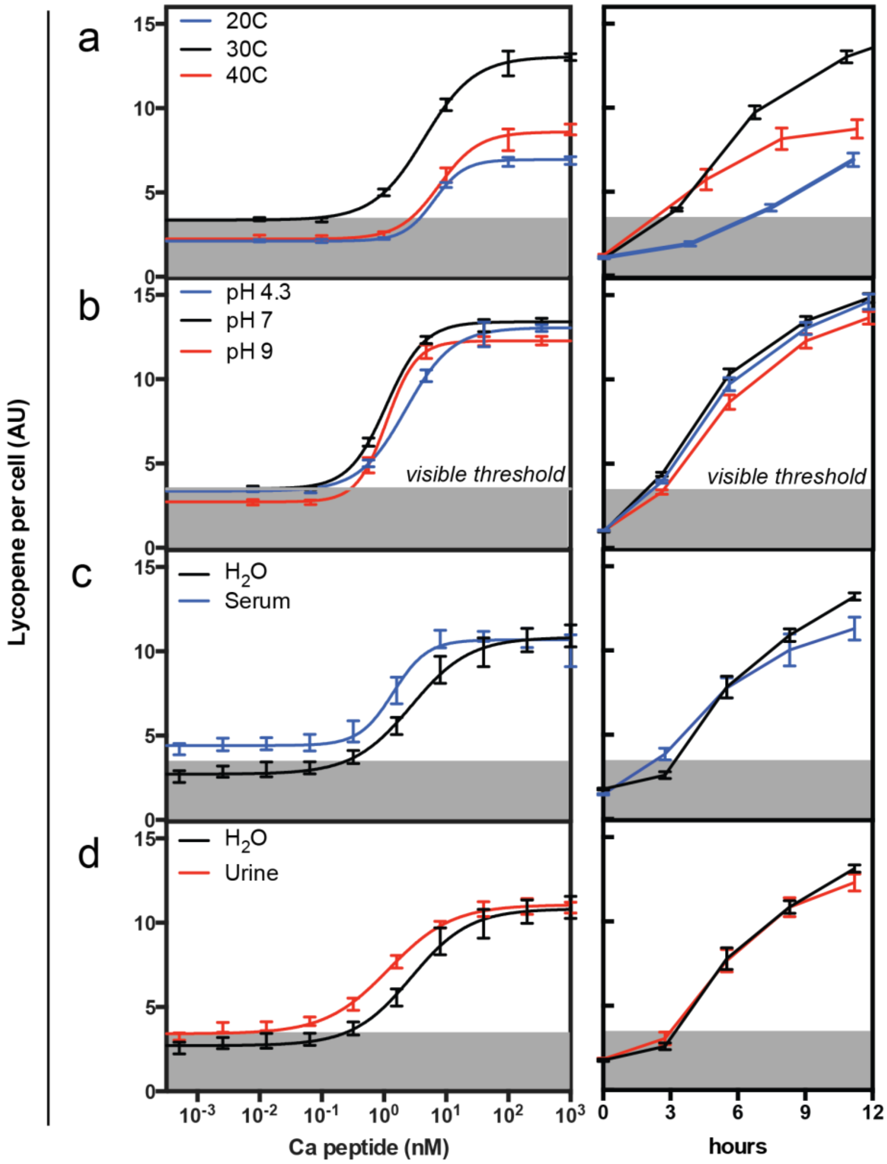

	LoD (nM)
20C	~ 10
30C	~ 1
40C	~ 10

	LoD (nM)
pH 4.3	~ 1
pH 7	~ 1
pH 9	~ 10

	LoD (nM)
H_2O	~ 0.3
Serum	~ 8

	LoD (nM)
H_2O	~ 0.3
Urine	~ 0.3

fig. S2. *C. albicans* biosensor robustness in liquid culture. Dose-response and time-course data for S. cerevisiae strain carrying *C. albicans* Ste2 receptor (Ca.Ste2) under different conditions: (**A**) - temperature, (**B**) - pH, (**C**) - 50% human serum and (**D**) - 50% human urine. Lycopene yield was determined by absorbance after 9 hours. All experiments were performed using 1 µM synthetic peptide. The limit of detection (LoD, lowest peptide concentration producing significant signal over background, ** $P \leq 0.01$) is shown for each sample conditions. N=3.

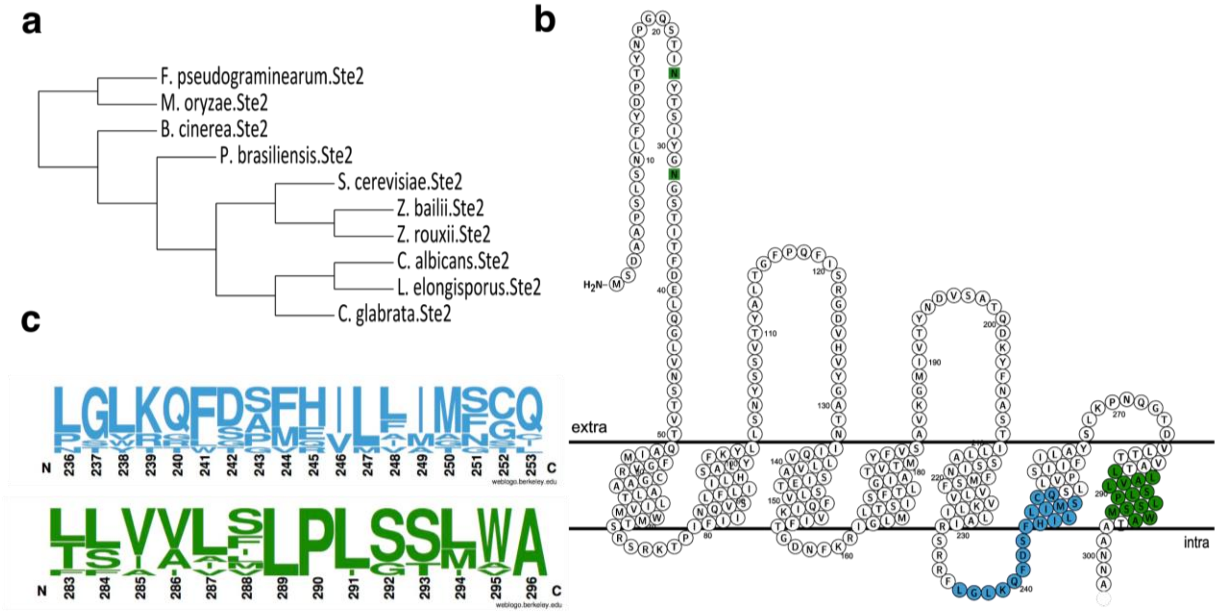

fig. S3. Sequence analysis of fungal mating receptors. (**A**) Phylogenetic tree built using the mating receptor protein sequence (Ste2) for the following fungal pathogens: *S. cerevisiae* (Sc), *Candida albicans* (Ca), *Candida glabrata* (Cg), *Paracoccidioides brasiliensis* (Pb), *Lodderomyces elongisporus* (Le), *Botrytis cinerea* (Bc), *Fusarium graminearum* (Fg), *Magnaporthe oryzae* (Mo), *Zygosaccharomyces bailii* (Zb), and *Zygosaccharomyces rouxii* (Zr). Tree was generated using phylogeny.fr with default settings with receptor sequences noted in table S1. (**B**) Wild type S. cerevisiae Ste2 mating receptor (UniProtKB: D6VTK4) visualized using Protter software (*52*). Residues suggested to mediate signal transduction and interactions with the downstream G-protein are highlighted in color (*53*, *54*). (**C**) Amino acid frequency for residues highlighted in B across all shown species. Protein sequences were aligned using MUSCLE (*37*) and amino acid frequency was analyzed using weblogo (*55*) for residues corresponding to position 236-253 (blue) and 283-296 (green) of the *S. cerevisiae* Ste2 protein sequence.

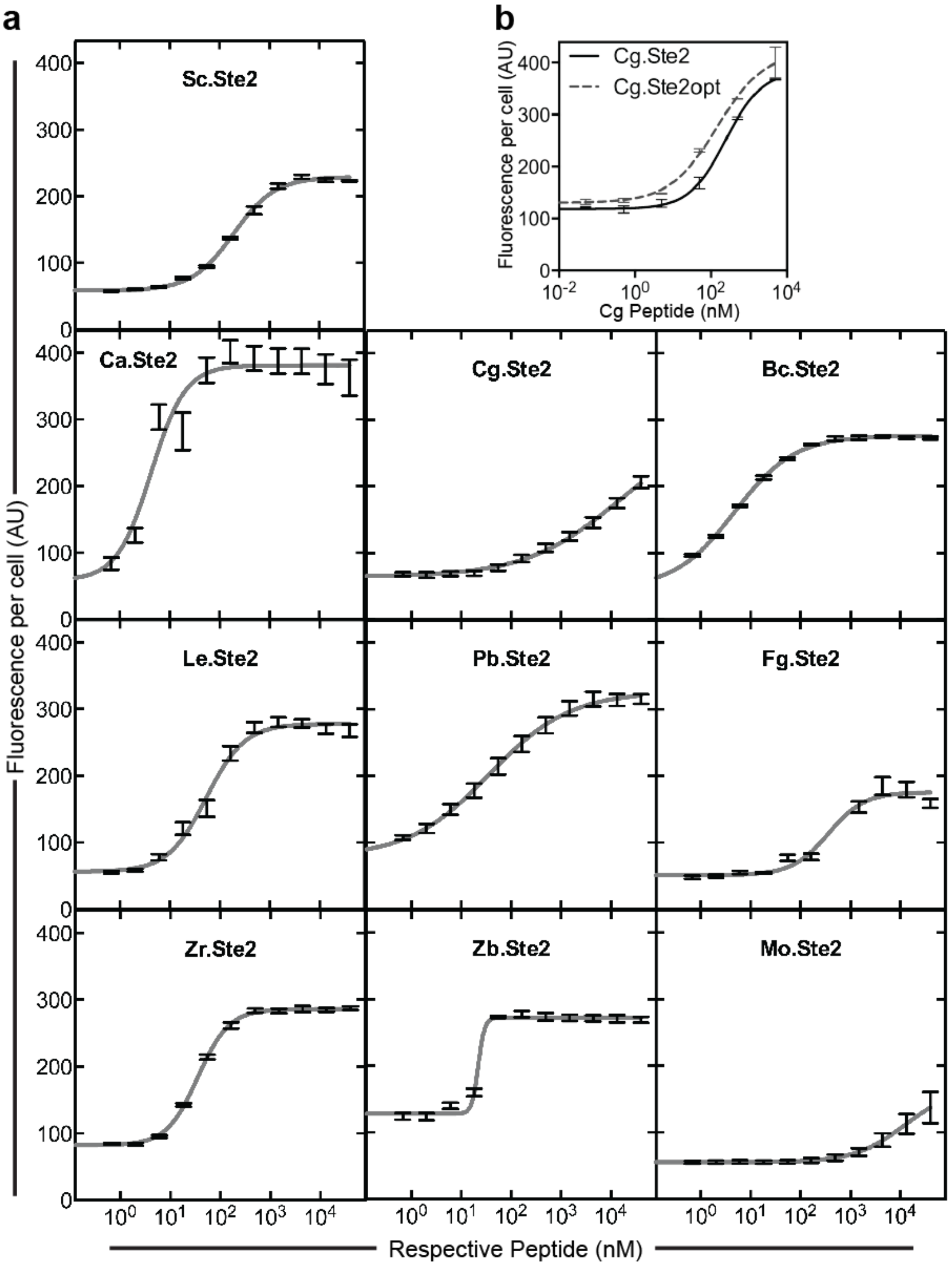

fig. S4. Dose-response curves for fungal mating receptors. (**A**) Heterologous mating receptors from the indicated fungal strains were engineered to replace the endogenous *S. cerevisiae* Ste2 mating receptor. Each strain was tested with its cognate synthetic fungal peptide. Receptor activation was monitored by activation of mCherry fluorescent reporter gene under the control of *FUS1* pheromone-inducible promoter after 12 hours and EC_{50} value was measured (see Fig. 3B). (**B**) Dose response curve for *C. glabrata* wild type mating receptor (Cg.Ste2) and codon-optimized receptor (Cg.Ste2opt).

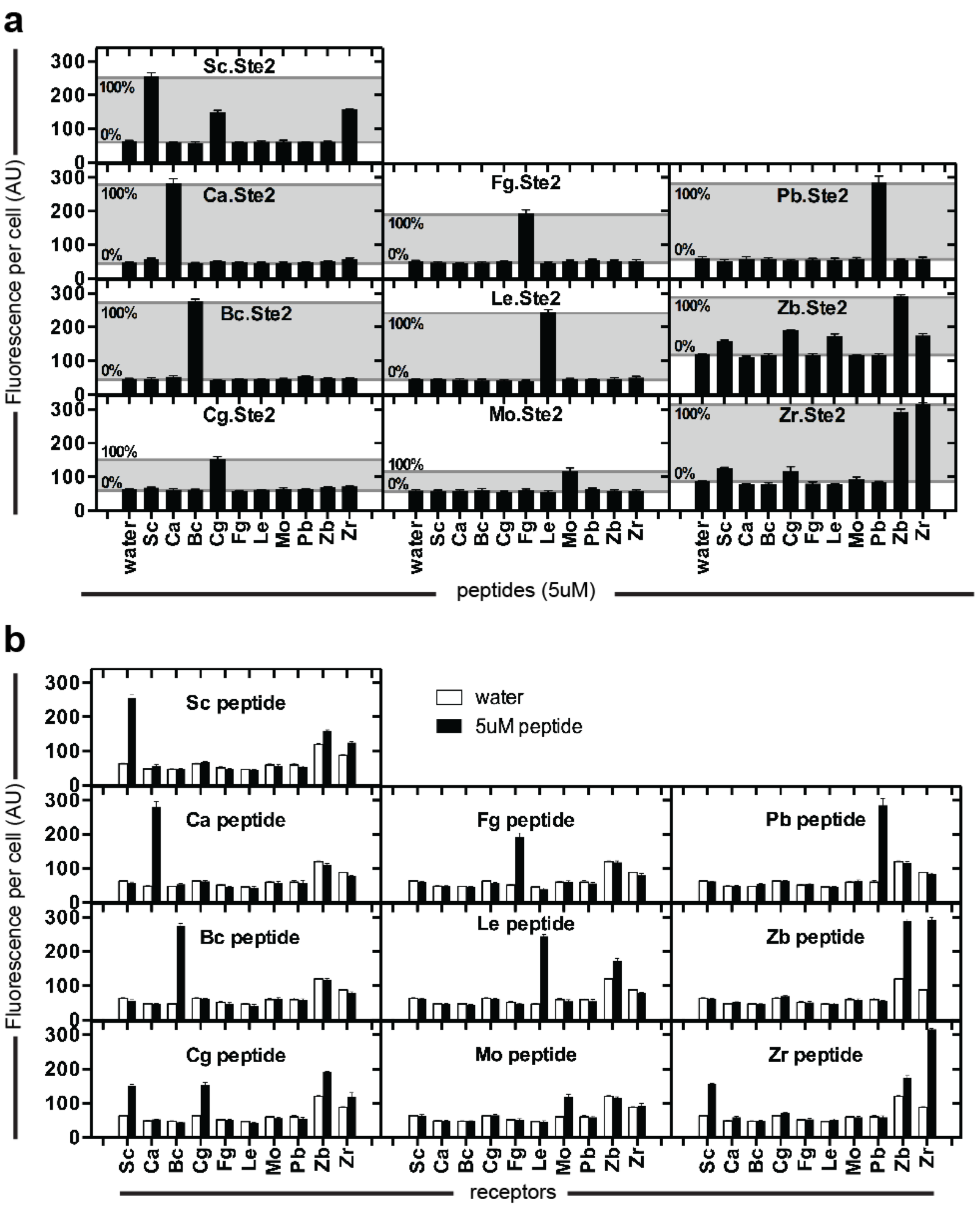

fig. S5. Specificity of fungal mating receptors. (**A**) Heterologous receptors (‘*species*.Ste2’) were induced with 5 µM of the indicated fungal mating peptide. mCherry fluorescence was measured after 9 hours. Basal (0%) and maximal (100%) fluorescence used to generate Fig. 3C indicated in grey. (**B**) Data as in A. Activation of heterologous mating receptors shown here grouped by mating peptide.

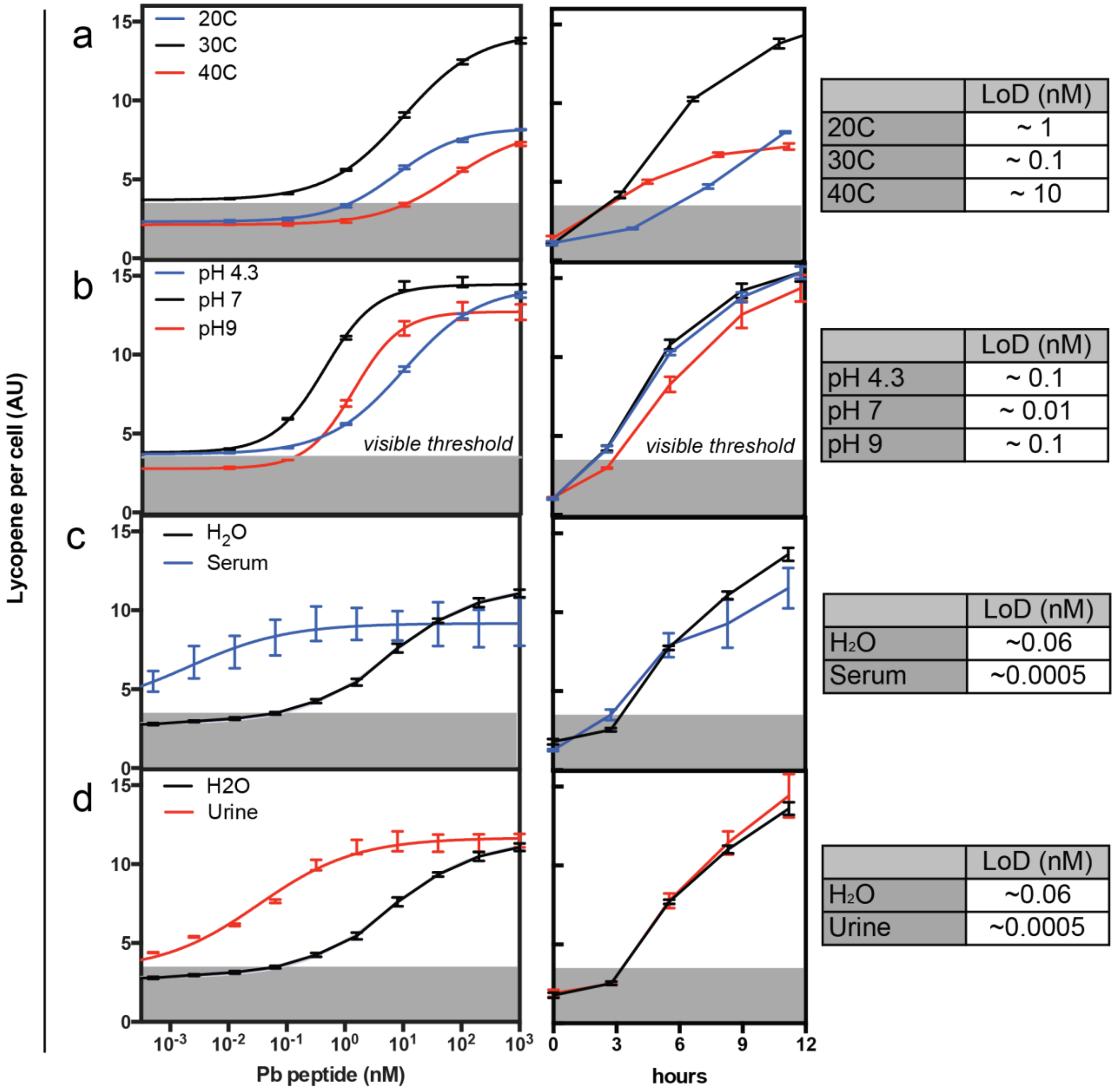

fig. S6. ***P. brasiliensis*** **biosensor characterization in liquid culture.** Dose-response and time-course data shown for *S. cerevisiae* strain carrying *P. brasiliensis* Ste2 receptor (Ca.Ste2) under different conditions: (**A**) - temperatures, (**B**) - pH, (**C**) - 50% human serum and (**D**) - 50% human urine. Lycopene yield was determined by absorbance after 9 hours. All experiments were performed using 1 µM synthetic peptide. The limit of detection (LoD, lowest peptide concentration producing significant signal over background, ** $P \leq 0.01$) is shown for each sample conditions. N=3.

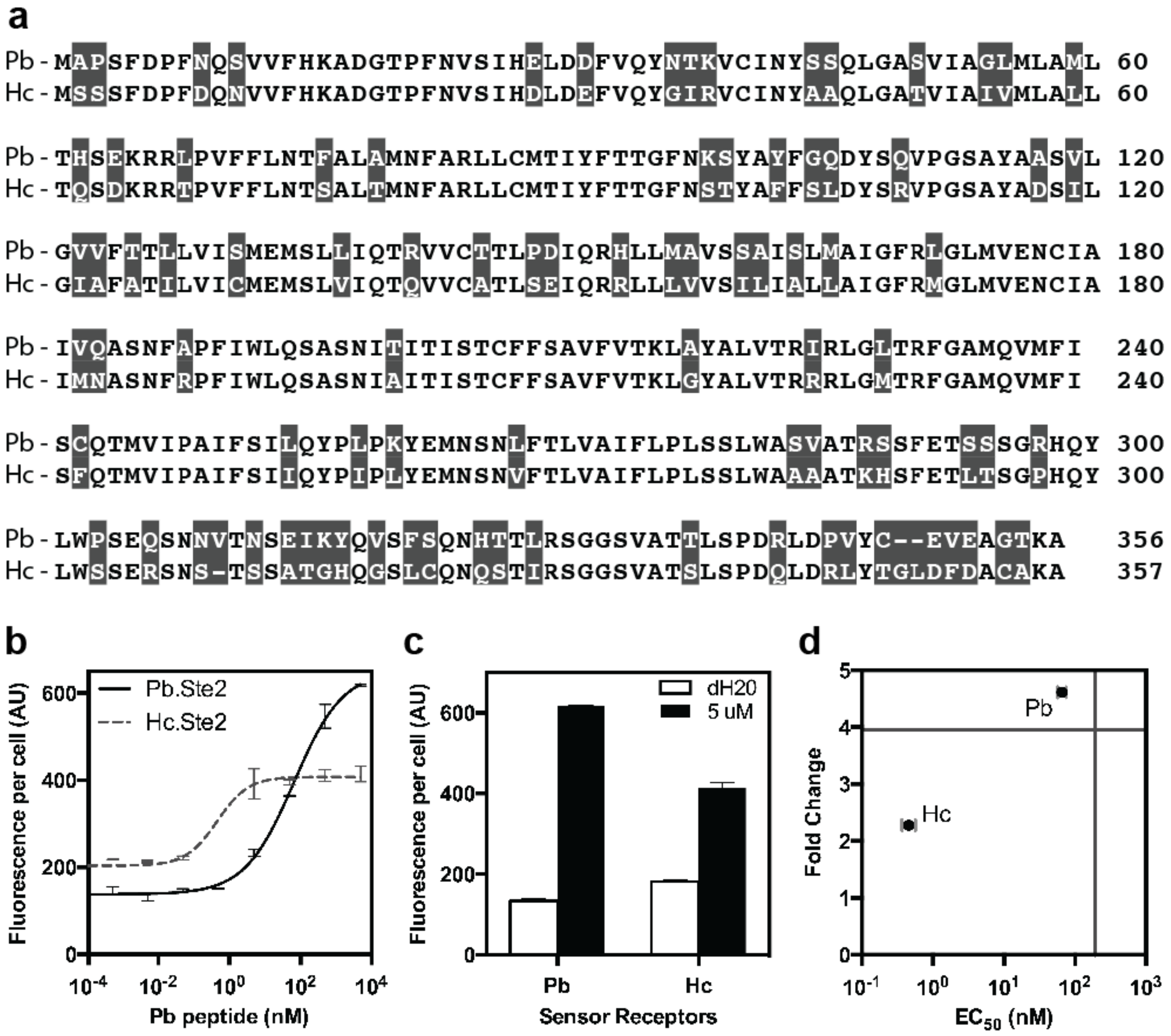

fig. S7. Comparison of mating receptors from human pathogens *P. brasiliensis* and *H. capsulatum*. (**A**) Protein sequence comparison of the *P. brasiliensis* (Pb.Ste2) and *H. capsulatum* (Hc.Ste2) receptors. Positions that differ highlighted in grey. (**B**) Dose response curve using Pb.Ste2 and Hc.Ste2 receptors cloned in *S. cerevisiae* and induced with the common cognate ligand (table S1). Measurement was taken after 12 hours. All measurements were performed in duplicate. (**C**) Comparison of basal (dH_2O) and maximum (5 µM) activation level for Pb and Hc mating receptor using the same synthetic ligand, as shown in B. (**D**) Comparison of Pb.Ste2 and Hc.Ste2 receptors fold-activation and EC_{50} values calculated from panel B. Grey cross lines mark the equivalent values for *S. cerevisiae* wild type mating receptor Ste2 activated by its own cognate peptide. While Hc.Ste2 exhibited higher sensitivity to the common mating peptide than Pb.Ste2, it also had higher basal level and lower maximal activation making it less effective for detection using the visible lycopene readout.

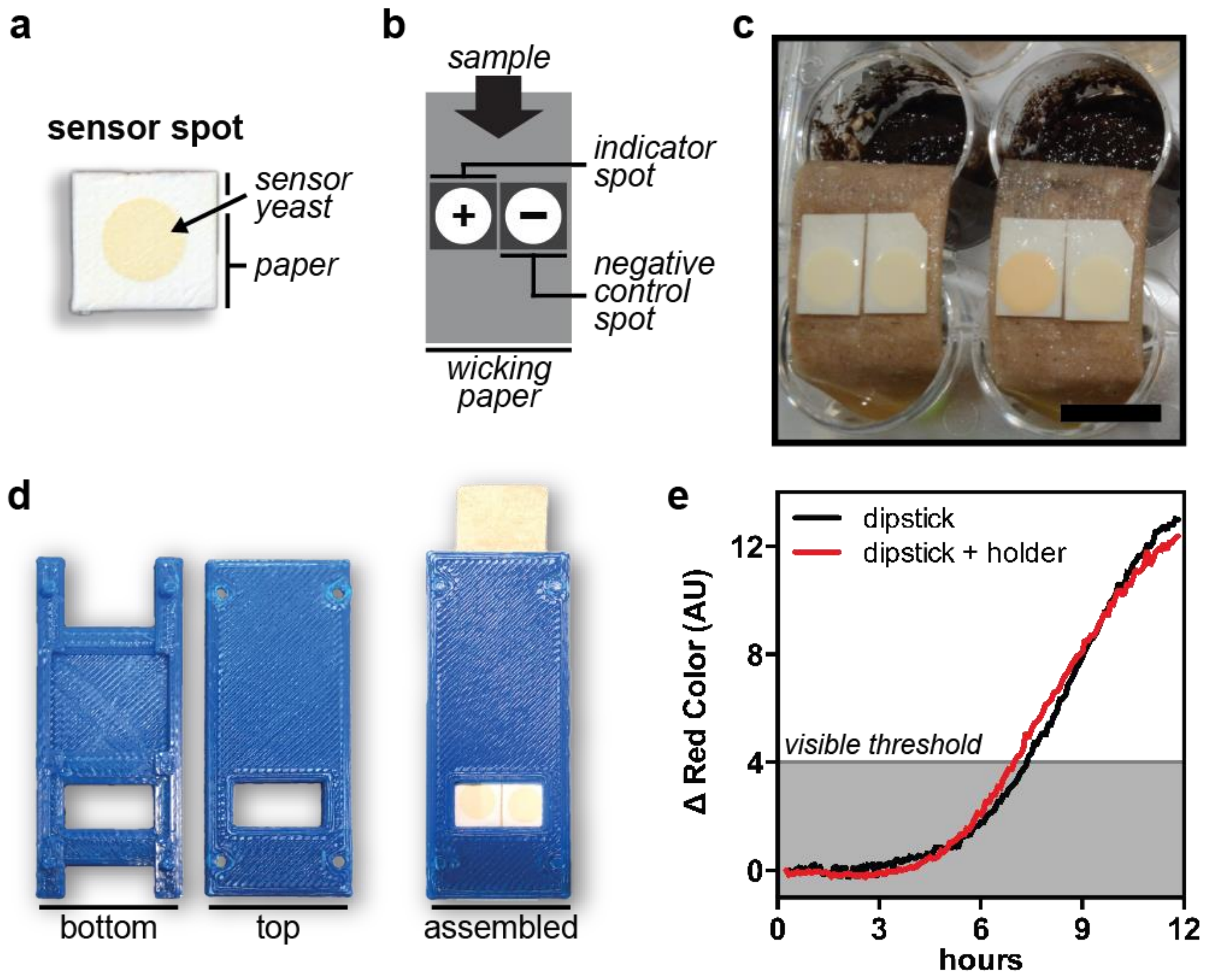

fig. S8. Paper-based dipstick assay. (**A**) Engineered *S. cerevisiae* biosensor cells spotted on paper are the only active component required for the dipstick assay. Spot diameter - 5 mm. (**B**) Dipstick assay includes two spots, indicator biosensor strain and control strain, placed on top of a strip of paper towel that acts as wicking paper. The indicator biosensor spot detects the target ligand and the negative control spot contains a strain with an off-target receptor. This design enables easy visual interpretation of the results as well as quantification by calculating the difference in the pixel color values between the two spots (see Supplementary Methods). (**C**) Representative photograph of the dipstick for detection of the fungal pathogen *P. brasiliensis* in soil. Left - no mating peptide in soil. Right - mating peptide added to soil. Scale bar - 1 cm. (**D**) A simple plastic holder was designed to enable easy use of the dipstick assay. Thin black bars - 2 cm. (**E**) Dipstick holder does not affect biosensor performance as shown by time course measurement of the *P. brasiliensis* dipstick test response using 1 µM cognate peptide.

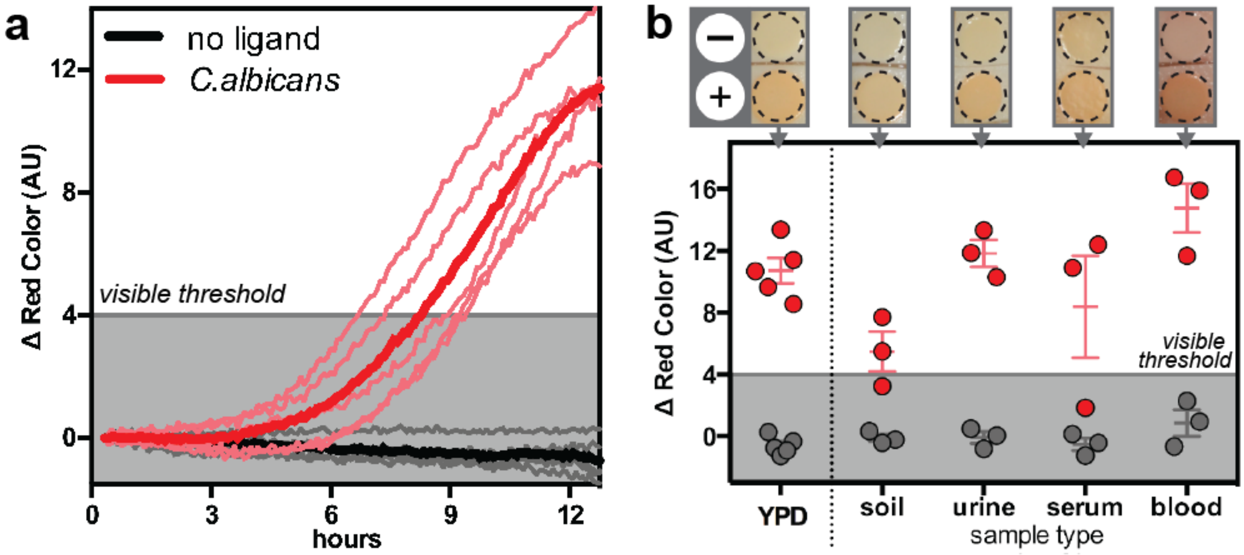

fig. S9. Detection of *C. albicans* using dipstick assay. (**A**) Quantitative analysis of color change using dipstick assay, as scored by time-lapse photography for detection of synthetic *C. albicans* mating peptide (1 µM in YPD media). Individual runs in light colors, average response shown in dark color. Grey shading indicates visible threshold. (**B**) Dipstick assay successfully reports *C. albicans* mating peptide in complex samples. Liquid samples were supplemented with 1 µM synthetic *C. albicans* mating peptide and scored as in A. YPD - media only, Soil - standard potting soil, Urine - 50% pooled human urine, Serum - 50% human serum, Blood - 2% whole blood. All experiments performed using 1 µM peptide and supplemented with YPD media.

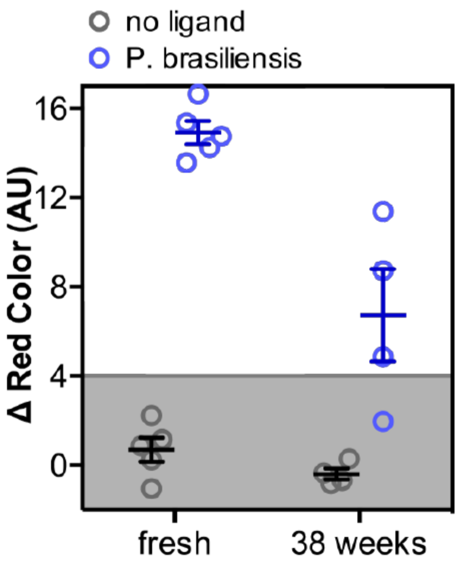

fig. S10. Long-term stability of paper-based dipsticks stored at room temperature. The paper-based dipsticks were air-dried without additives and stored for 38 weeks at room temperature under conditions analogous to commercial yeast products (in sealed, dark pouches flushed with an inert atmosphere). The stored dipsticks successfully report on *P. brasiliensis* mating peptide after rehydration directly with the sample. Individual runs in light colors, average response shown in dark color. Grey shading indicates visible threshold. All experiments performed using 1 μM peptide in YPD media and quantified at 15 hours.

table S1. Fungal pathogen peptides and receptor genes used in this study.

Species	Association	Pathogenic Target	Synthetic Peptide Sequence	Receptor UniProt ID	Receptor Source
Saccharomyces cerevisiae	Baker's yeast	-	WHWLQLKPGQPMY	D6VTK4	ATCC 200895
Candida glabrata	Candidiasis	Human	WHWVRLRKGQGLF	Q6FLY8	ATCC 2001
Candida albicans	Candidiasis	Human	GFRLTNFGYFEPG	Q59Q04	ATCC MYA-2876
Lodderomyces elongisporus	Candidiasis	Human	WMWTRYGRFSPV	A5E1D9	ATCC 11503
Paracoccidioides brasiliensis (lutzii)	Paracoccidioido-mycosis	Human	WCTRPGQGC	C1GFU7	Plasmid pLPreB(*30*)
Botrytis cinerea (Botryotinia fuckeliana)	Gray mold	Plants	WCGRPGQPC	G2YE05	codon-optimized synthetic DNA
Fusarium graminearum (Gibberella zeae)	Wheat head blight	Plants	WCWWKGQPCW	I1RG07	codon-optimized synthetic DNA
Magnaporthe oryzae	Rice blast	Plants	QWCPRRGQPCW	G4MR89	codon-optimized synthetic DNA
Zygosaccharomyces bailii	Spoilage	Food spoilage	HLVRLSPGAAMF	S6EXB4	codon-optimized synthetic DNA
Zygosaccharomyces rouxii	Spoilage	Food spoilage	HFIELDPGQPMF	C5DX97	ATCC 2623
Histoplasma capsulatum	Histoplasmosis	Human	WCTRPGQGC	C0NQ16	codon-optimized synthetic DNA

table S2. Strains used in this study. All strains are *S. cerevisiae* unless otherwise noted. Strains were generated in this study except where a source is noted. The nomenclature "ReRec[N]::" refers to expression modules inserted in the Nth round of reiterative recombination at the acceptor site located in the HO locus (*32*).

Strain	Genotype	Comments
FY251	*MAT*a *his3-Δ200, leu2-Δ1 trp1-Δ63*, *ura3-52*	ATCC 96098
BY4733	*MATa his3Δ200 leu2Δ0 met15Δ0 trp1Δ63 ura3Δ0*	ATCC 200895
LW2591	BY4733 *MATa-inc HOΔ::ReRec*	Reiterative Recombination acceptor strain (*32*)
LW2671	BY4733 derivative overexpressing *CrtEBI*	Constitutive lycopene producing strain (*40*)
yMJ105	LW2591 *sst2-Δ far1-Δ*	Parental biosensor strain
Fluorescence Readout Strains		
yMJ183	yMJ105 *ste2-Δ fus1Δ::pFUS1-HIS3-tHIS3* ReRec[1]::*pFUS1-yCherry-tACT1*	Receptor-less fluorescence biosensor strain
yMJ281	yMJ183 + pMJ093	*S. cerevisiae* biosensor
yMJ282	yMJ183 + pMJ090	*C. albicans* biosensor
yMJ284	yMJ183 + pMJ095	*B. cinerea* biosensor
yMJ285	yMJ183 + pMJ096	*C. glabrata* biosensor
yMJ286	yMJ183 + pMJ097	*F. graminearum* biosensor
yMJ288	yMJ183 + pMJ099	*L. elongisporous* biosensor
yMJ289	yMJ183 + pMJ100	*M. oryzea* biosensor
yMJ290	yMJ183 + pMJ101	*P. brasiliensis* biosensor
yMJ294	yMJ183 + pMJ105	*Z. bailii* biosensor
yMJ295	yMJ183 + pMJ106	*Z. rouxii* biosensor
yMJ312	yMJ183 + pMJ117	*H. capsulatum* biosensor
yJM06	yMJ183 + pJM13	Codon-optimized *C. glabrata* biosensor
Lycopene Biosensor Strains		
yMJ116	yMJ105 ReRec[1]::*pTEF1-CrtE-tADH1*-(*CrtB-pPGK1*,rev)	Lycopene null strain
yMJ118	yMJ105 ReRec[1]::*pTEF1-CrtE-tADH1*-(*CrtB-pPGK1*,rev) ReRec[2]::*pFUS1-CrtI-tACT1*	Unoptimized lycopene biosensor Lyco-1
yMJ151	yMJ118 + pMJ006	"+ 2X CrtI" intermediate
yMJ152	yMJ118 + pMJ009	"+ tHMG1" intermediate
yMJ165	yMJ118 + pMJ012	"+ FAD1" intermediate
yMJ251	yMJ105 *met15Δ::pFUS1-CrtI-tACT1-MET15* ReRec[1]::*pTEF1-CrtE-tADH1*-(*CrtB-pPGK1*,rev) ReRec[2]::*pFUS1-CrtI-tACT1* ReRec[3]::*pTDH3-FAD1-tPGK1*	Optimized lycopene biosensor Lyco-2 (Sc biosensor)
yMJ258	yMJ251 ste2Δ::pTDH3-Pb.Ste2-tSTE2	Pb biosensor
yMJ260	yMJ251 ste2Δ::pTDH3-Ca.Ste2-tSTE2	Ca biosensor
Strains Used to Generate Pathogen and Control Supernatants		
W303-1B	*MATα leu2-3,112 trp1-1 can1-100 ura3-1 ade2-1 his3-11,15*	ATCC 201238
FY250	*MATα his3-Δ200, leu2-Δ1 trp1-Δ63*, *ura3-52*	(*50*)
GC75	*Candida albicans*, MTLα/MTLα	Genebank assembly number GCA_000773735.1 (*46*)
ySB36	*Candida albicans*, MTLa/MTLα	Clinical isolate obtained from A-C. Uhlemann, mating loci

		(MTL) were genotyped by PCR
ySB45	*Candida albicans*, MTLα/MTLα	sorbose selected isolate, derivative of isolate ySB36, MTL were genotyped by PCR
Pb01	*Paracoccidioides lutzii*, MAT1-1	Supernatant prepared by Prof. Fernando Rodrigues (*44*)
Pb18	*Paracoccidioides brasiliensis*, MAT1-2	Supernatant prepared by Prof. Fernando Rodrigues (*44*)
Hc01	*Histoplasma capsulatum*, NAm2	Supernatant prepared by Prof. Chad Rappleye (*42*)
Hc06	*Histoplasma capsulatum*, NAm1	Supernatant prepared by Prof. Chad Rappleye (*42*)

table S3. Plasmids used in this study. Plasmids were generated in this study except where a source is noted.

Plasmid	Construct Details	Comments
pSC203	*Erwinia herbicola* CrtEBI	Kind gift from Gregory Stephanopoulos
yEpGAP-Cherry	Yeast codon-optimized mCherry	Kind gift from Neta Dean (*39*)
pLPreB	*P. brasiliensis* mating receptor	Kind gift from Fernando Rodrigues (*30*)
pMJ006	pRS416, pFUS1-CrtI-tACT1	Pheromone inducible CrtI
pMJ009	pRS416, pTDH3-tHMG1-tCYC1	Overexpressed truncated HMG1
pMJ012	pRS416, pTDH3-FAD1-tCYC1	Overexpressed FAD1
pMJ090	pRS416, pTDH3-Ca.Ste2-tSTE2	Overexpressed *C. albicans* Ste2 homologue
pMJ093	pRS416, pTDH3-Sc.Ste2-tSTE2	Overexpressed wild type Ste2
pMJ095	pRS416, pTDH3-Bc.Ste2-tSTE2	Overexpressed *B. cinerea* Ste2 homologue
pMJ096	pRS416, pTDH3-Cg.Ste2-tSTE2	Overexpressed *C. glabrata* Ste2 homologue
pMJ097	pRS416, pTDH3-Fg.Ste2-tSTE2	Overexpressed *F. graminearum* Ste2 homologue
pMJ099	pRS416, pTDH3-Le.Ste2-tSTE2	Overexpressed *L. elongisporus* Ste2 homologue
pMJ100	pRS416, pTDH3-Mo.Ste2-tSTE2	Overexpressed *M. oryzea* Ste2 homologue
pMJ101	pRS416, pTDH3-Pb.Ste2-tSTE2	Overexpressed *P. brasiliensis* Ste2 homologue
pMJ105	pRS416, pTDH3-Zb.Ste2-tSTE2	Overexpressed *Z. bailii* Ste2 homologue
pMJ106	pRS416, pTDH3-Zr.Ste2-tSTE2	Overexpressed *Z. rouxii* Ste2 homologue
pMJ117	pRS416, pTDH3-Hc.Ste2-tSTE2	Overexpressed *H. capsulatum* Ste2 homologue
pJM13	pRS416, pTDH3-Cg.Ste2opt-tSTE2	Overexpressed codon-optimized *C. glabrata* Ste2 homologue

table S4. List of expression modules constructed in this study. Promoters and terminators in upper case, open reading frames (ORFs) in lower case.

Description	Sequences
Receptor Expression Module	
pTDH3-*species*.Ste2-tSTE2	AGTTTATCATTATCAATACTGCCATTTCAAAGAATACGTAAATAATTAATAGTAGTGATTTTCCTAACTTTATT<br>TAGTCAAAAAATTAGCCTTTTAATTCTGCTGTAACCCGTACATGCCCAAAATAGGGGGCGGGTTACACAGAAT<br>ATATAACATCGTAGGTGTCTGGGTGAACAGTTTATTCCTGGCATCCACTAAATATAATGGAGCCCGCTTTTTA<br>AGCTGGCATCCAGAAAAAAAAAGAATCCCAGCACCAAAATATTGTTTTCTTCACCAACCATCAGTTCATAGGT<br>CCATTCTCTTAGCGCAACTACAGAGAACAGGGGCACAAACAGGCAAAAAACGGGCACAACCTCAATGGAGTG<br>ATGCAACCTGCCTGGAGTAAATGATGACACAAGGCAATTGACCCACGCATGTATCTATCTCATTTTCTTACAC<br>CTTCTATTACCTTCTGCTCTCTCTGATTTGGAAAAAGCTGAAAAAAAAAGGTTGAAACCAGTTCCCTGAAATTAT<br>TCCCCTACTTGACTAATAAGTATATAAAGACGGTAGGTATTGATTGTAATTCTGTAAATCTATTTCTTAAACTT<br>CTTAAATTCTACTTTTATAGTTAGTCTTTTTTTTAGTTTTAAAACACCAAGAACTTAGTTTCGACGGATACTAGT<br>AAA***[receptor ORF]***CTCGAGACGGCTTTGAAAAAGTAATTTCGTGACCTTCGGTATA<br>AGGTTACTACTAGATTCAGGTGCTCATCAGATGCACCACATTCTCTATAAAAAAAAATGGTATCTTTCTTATTT<br>GATAATATTTAAACTCCTTTACATAATAAACATCTCGTAAGTAGTGGTAGAAACCACCTTTGCTTTTACGAGTT<br>CAAGCTTTTTTCTTGCCATGATCTAGAACTCTCAGGCAATATATACAGTTAATCTTTTTTTACTGGGTTGTAGTT<br>CTAATGTATTGTTTCGAAAAATAGCAACCAGGCACA
Fluorescent Reporter Module (yMJ183)	
pFUS1-yCherry-tACT1	TACCATGTGGACCCTTTCAAAACAGAGTTGTATCTCTGCAGGATGCCCTTTTTGACGTATTGAATGGCATAATT<br>GCACTGTCACTTTTCGCGCTGTCTCATTTTGGTGCGATGATGAAACAAACATGAAACGTCTGTAATTTGAAAC<br>AAATAACGTAATTCTCGGGATTGGTTTTATTTAAATGACAATGTAAGAGTGGCTTTGTAAGGTATGTGTTGCTC<br>TTAAAATATTTGGATACGACATCCTTTATCTTTTTTCCTTTAAGAGCAGGATATAAGCCATCAAGTTTCTGAAA<br>ATCAAAatggtttcaaaaggtgaagaagataatatggctattattaaagaatttatgagatttaaagttcatatggaaggttcagttaatggtcatgaatttgaaattgaaggtgaagg<br>tgaaggtagaccatatgaaggtactcaaactgctaaattgaaagttactaaaggtggtccattaccatttgcttgggatattttgtcaccacaatttatgtatggttcaaaagcttatgttaaa<br>catccagctgatattccagattatttaaaattgtcatttccagaaggttttaaatgggaaagagttatgaattttgaagatggtggtgttgttactgttactcaagattcatcattacaagatggt<br>gaatttatttataaagttaaattgagaggtactaattttccatcagatggtccagttatgcaaaaaaaaactatgggttgggaagcttcatcagaaagaatgtatccagaagatggtgcttta<br>aaaggtgaaattaaacaaagattgaaattaaaagatggtggtcattatgatgctgaagttaaaactacttataaagctaaaaaaccagttcaattaccaggtgcttataatgttaatattaaa<br>ttggatattacttcacataatgaagattatactattgttgaacaatatgaaagagctgaaggtagacattcaactggtggtatggatgaattatataaataaTCTCTGCTTTTGTG<br>CGCGTATGTTTATGTATGTACCTCTCTCTCTATTTCTATTTTTAAACCACCCTCTCAATAAAATAAAAATAATA<br>AAGTATTTTTAAGGAAAAGACGTGTTTAAGCACTGACTTTATCTACTTTTTGTACGTTTTCATTGATATAATGT<br>GTTTTGTCTCTCCCTTTTCTACGAAAATTTCAAAAATTGACCAAAAAAAGGAATATATATACGAAAAACTATT<br>ATATTTATATATCATAGTGT
Lycopene Readout Modules (yMJ116, yMJ118, yMJ251)	
pTEF1-CrtE-tADH1-(CrtB-pPGK1, rev)	ATAGCTTCAAAATGTTTCTACTCCTTTTTTACTCTTCCAGATTTTCTCGGACTCCGCGCATCGCCGTACCACTTC<br>AAAACACCCAAGCACAGCATACTAAATTTCCCCTCTTTCTTCCTCTAGGGTGTCGTTAATTACCCGTACTAAAG<br>GTTTGGAAAAGAAAAAAGAGACCGCCTCGTTTCTTTTTCTTCGTCGAAAAAGGCAATAAAAATTTTTATCACG<br>TTTCTTTTTCTTGAAAATTTTTTTTTTGATTTTTTTCTCTTTCGATGACCTCCCATTGATATTTAAGTTAATAAA<br>CGGTCTTCAATTTCTCAAGTTTCAGTTTCATTTTTCTTGTTCTATTACAACTTTTTTTACTTCTTGCTCATTAGAA<br>AGAAAGCATAGCAATCTAATCTAAGTTTTAATTACAAAatggtttctggttcgaaagcaggagtatcacctcatagggaaatcgaagtcatgaga<br>cagtccattgatgaccacttagcaggattgttgccagaaacagattcccaggatatcgttagccttgctatgagagaaggtgttatggcacctggtaaacgtatcagacctttgctgatgt<br>tacttgctgcaagagacctgagatatcagggttctatgcctacactactggatctagcttgtgctgttgaactgacacatactgcttccttgatgctggatgacatgccttgtatggacaatg<br>cggaacttagaagaggtcaaccaacaacccacaagaaattcggagaatctgttgccattttggcttctgtaggtctgttgtcgaaagcttttggcttgattgctgcaactggtgatcttcca<br>ggtgaaaggagagcacaagctgtaaacgagctatctactgcagttggtgttcaaggtctagtcttaggacagttcagagatttgaatgacgcagctttggacagaactcctgatgctatc<br>ctgtctacgaaccatctgaagactggcatcttgttctcagctatgttgcaaatcgtagccattgcttctgcttcttcaccatctactagggaaacgttacacgcattcgcattggactttggtc<br>aagcctttcaactgctagacgatttgagggatgatcatccagagacaggtaaagaccgtaacaaagacgctggtaaaagcactctagtcaacagattgggtgctgatgcagctagac<br>agaaactgagagagcacattgactctgctgacaaacacctgacatttgcatgtccacaaggaggtgctataaggcagtttatgcacctatggtttggacaccatcttgctgattggtctcc<br>agtgatgaagatcgcctaaGCGAATTTCTTATGATTTATGATTTTTATTATTAAATAAGTTATAAAAAAAATAAGTGTATA<br>CAAATTTTAAAGTGACTCTTAGGTTTTAAAACGAAAATTCTTATTCTTGAGTAACTCTTTCCTGTAGGTCAGGT<br>TGCTTTCTCAGGTATAGCATGAGGTCGCTCttagacaggtctttgccataaaccagcaggtcttggtgtaactctggttgttttggcacgaatgacttgtcctg<br>gtgcagccataagcatcgcaatcttctctcctttgctagtgtgttgacgtctatcccaagcagaaccacctgcagcttttaccttgatgccaatctctctgtagacagatcttgcagtagcta<br>tagcccaagcacatctaggtggtagatcatgcaatccagcttgacttgagatgtaatagggttcagcagcgtctatcagcctttcagcaactcttgctaatgcagctctgttctctcttgca<br>gcgtaattctcaggagttagaccagcatcttgcaaccattcagcaggtagatagcatctgtcaatagctgcatcgtcgataatatctctcgcgatgtttgtcagctgaaaagccaaaccta<br>gatcacaagctctgtccaaaaccctttcgtctcttacacccattactcttgccatcatcaaaccaactactccagcaacatggtagcagtatctcaaggtgtcttcaaaggtcacgtaacga<br>gtttgagcaacatccattgcgaaaccatccaagtgatcaagtgccattcttggcgtaataccgtgtgttagtgcaacttcttggaatgcagcaaaagcaggatcttgcatctcagcacctt<br>caaaagctgcaagtgtaagcgttctcaatctagccaatctctgagtagcctcttcttctgcagcagcttcagatgcgaaaccatgtgtctggtcatctataacgtcatcacagtgtctacac<br>caagtgtacagcataagcactgatcttctagtagctgggtcaaacagtttagctgctgtagcaaaggacttggaaccattagccatcgtttgagtagcatgatccaacaaaggtggttga<br>ctcatTGTTTTATATTTGTTGTAAAAAGTAGATAATTACTTCCTTGATGATCTGTAAAAAAGAGAAAAAGAAAGC<br>ATCTAAGAACTTGAAAAACTACGAATTAGAAAAGACCAAATATGTATTTCTTGCATTGACCAATTTATGCAAG<br>TTTATATATATGTAAATGTAAGTTTCACGAGGTTCTACTAAACTAAACCACCCCCTTGGTTAGAAGAAAAGAG<br>TGTGTGAGAACAGGCTGTTGTTGTCACACGATTCGGACAATTCTGTTTGAAAGAGAGAGAGTAACAGTACGAT<br>CGAACGAACTTTGCTCTGGAGATCACAGTGGGCATCATAGCATGTGGTACTAAACCCTTTCCCGCCATTCCAG<br>AACCTTCGATTGCTTGTTACAAAACCTGTGAGCCGTCGCTAGGACCTTGTTGTGTGACGAAATTGGAAGCTGC<br>AATCAATAGGAAGACAGGAAGTCGAGCGTGTCTGGGTTTTTTCAGTTTTGTTCTTTTTGCAAACAAATCACGA<br>GCGACGGTAATTTCTTTCTCGATAAGAGGCCACGTGCTTTATGAGGGTAACATCAATTCAAGAAGGAGGGAA<br>ACACTTCCTTTTTCTGGCCCTGATAATAGTATGAGGGTGAAGCCAAAATAAAGGATTCGCGCCCAAATCGGCA<br>TCTTTAAATGCAGGTATGCGATAGTTCCTCACTCTTTCCTTACTCACGAGTAATTCTTGCAAATGCCTATTATG<br>CAGATGTTATAATATCTGTGCGTCTTGAGTTGAAGTCAGGAATCTAAAATAAAAATTAAGGTTAATAAAAAGA<br>GGAAAGAAAAAAAAATTAATCGATTTACAGAAACTTGCACACTAAAAATACACAACTAAAAGCAATTACAGT<br>ATGGGAAGTCATCGACGTTATCTCTACTATAGTATATTATCATTTCTATTATTATCCTGCTCAGTGGTACTTGC

	AAAACAAGATAAGACCCCATTCTTTGAAGGTACTTCTTCGAAAAATTCGCGTCT
pFUS1-CrtI-tACT1	TACCATGTGGACCCTTTCAAAACAGAGTTGTATCTCTGCAGGATGCCCTTTTTGACGTATTGAATGGCATAATT<br>GCACTGTCACTTTTCGCGCTGTCTCATTTTGGTGCGATGATGAAACAAACATGAAACGTCTGTAATTTGAAAC<br>AAATAACGTAATTCTCGGGATTGGTTTTATTTAAATGACAATGTAAGAGTGGCTTTGTAAGGTATGTGTTGCTC<br>TTAAAATATTTGGATACGACATCCTTTATCTTTTTTCCTTTAAGAGCAGGATATAAGCCATCAAGTTTCTGAAA<br>ATCAAAatgaagaaaaccgtagtgattggtgcaggttttggtggtttagctttggctatacgtctacaagctgcaggtattcctacagtgctattggagcaaagagacaaaccagg<br>aggaagagcttatgtttggcacgatcaaggctttacctttgatgctggtcctacagtcatcactgatcctactgcattggaagctttgttcaccttagctggtagaagaatggaagattatgt<br>ccgtctattgcctgtcaagccgttttacagattgtgttgggaatctggtaaaaccctagattacgccaatgacagtgctgaactagaagctcagattacgcagtttaatcccagagatgtc<br>gaaggttacaggagattccttgcctattcccaagctgttttccaagagggttatcttcgtttgggttcagttccattcctgtcctttagggatatgcttagagcaggtcctcagttgttgaagct<br>acaagcatggcaaagtgtgtatcagtctgtttcgagatttatcgaggatgaacatctgagacaagcattctcattccacagtcttctagttggaggtaatccctttaccacatcgagcatat<br>atacgttgattcacgctttggaaagagaatggggagtttggtttcctgaaggtggaacaggtgctttggttaatggtatggtgaagctattcacggatttgggtggagaaatagagctga<br>atgcaagagtggaagaacttgttgtagcagacaacagagtctcacaagttagacttgctgatggtaggatcttcgatacagatgctgtagcttcaaacgcagatgtagtgaacacttata<br>aaaagttgttgggacatcatcctgttggacaaaagagagcagctgctttggagaggaaatctatgagcaactcgttgtttgtcctttactttgggctgaatcaaccacactcacaactagc<br>tcatcacacaatctgctttggtcctagatacagagagctgatagatgaaattttcactggatctgctttagcagacgatttttccctgtacttgcattcaccatgtgttactgatccctctttagc<br>accacctggttgtgctagcttctatgtactagcacctgtaccacatttgggtaatgctccattagattgggcacaagaaggaccgaaattgagggataggatcttcgactatttggaagaa<br>cgttacatgccaggtttgagatctcagttggttacacagaggatattcacaccagctgattttcatgatactctagatgcgcatttaggtagcgctttttccattgagccacttttgacgcaaa<br>gtgcttggtttagaccacacaacagagattctgacattgccaatctgtacctagtaggtgcaggaactcatccaggagctggtattcctggagttgtagcttctgctaaagctactgctag<br>tctgatgatcgaggatttgcagtaaTCTCTGCTTTTGTGCGCGTATGTTTATGTATGTACCTCTCTCTCTATTTCTATTTTTAAA<br>CCACCCTCTCAATAAAATAAAAATAATAAAGTATTTTTAAGGAAAAGACGTGTTTAAGCACTGACTTTATCTA<br>CTTTTTGTACGTTTTCATTGATATAATGTGTTTTGTCTCTCCCTTTTCTACGAAAATTTCAAAAATTGACCAAAA<br>AAAGGAATATATATACGAAAAACTATTATATTTATATATCATAGTGT
pTDH3-FAD1-tPGK1	AGTTTATCATTATCAATACTCGCCATTTCAAAGAATACGTAAATAATTAATAGTAGTGATTTTCCTAACTTTAT<br>TTAGTCAAAAAATTAGCCTTTTAATTCTGCTGTAACCCGTACATGCCCAAAATAGGGGGCGGGTTACACAGAA<br>TATATAACATCGTAGGTGTCTGGGTGAACAGTTTATTCCTGGCATCCACTAAATATAATGGAGCCCGCTTTTTA<br>AGCTGGCATCCAGAAAAAAAAAAGAATCCCAGCACCAAAATATTGTTTTCTTCACCAACCATCAGTTCATAGGT<br>CCATTCTCTTAGCGCAACTACAGAGAACAGGGGCACAAACAGGCAAAAAACGGGCACAACCTCAATGGAGTG<br>ATGCAACCTGCCTGGAGTAAATGATGACACAAGGCAATTGACCCACGCATGTATCTATCTCATTTTCTTACAC<br>CTTCTATTACCTTCTGCTCTCTCTGATTTGGAAAAAGCTGAAAAAAAAAGGTTGAAACCAGTTCCCTGAAATTAT<br>TCCCCTACTTGACTAATAAGTATATAAAGACGGTAGGTATTGATTGTAATTCTGTAAATCTATTTCTTAAACTT<br>CTTAAATTCTACTTTTATAGTTAGTCTTTTTTTTAGTTTTAAAACACCAAGAACTTAGTTTCGAAGGATTCTAGA<br>ACTAGTAACatgcagttgagcaaggctgctgagatgtgttatgagataacaaactcttacttacacatagaccagaaatctcagataatagcaagtacacaagaagcgatacg<br>gttgacaagaaaatacttactaagtgaaatttttgtacgttggagtccactgaatggggaaatatcattctcgtacaacggaggaaaagattgccaggtattactactgttatatctgagttg<br>cttatgggaatatttcttcattaaggctcaaaattcccaattcgatttcgagtttcaaagcttccccatgcaaagacttccaactgttttcattgatcaagaagaaactttccctacattagaga<br>attttgtactggaaacctcagagcgatattgcctttccttatacgaatcacaaaggcaatctggtgcatcggtcaatatggcagacgcatttagagattttataaagatataccctgagacc<br>gaagctatagtgataggtattagacacacagacccatttggtgaagcattaaagcctattcaaagaacagattctaactggcctgattttatgaggttgcaacctctcttacactgggactt<br>aaccaatatatggagtttcttactgtattctaatgagccaatttgtggactatatggtaaaggtttcacatcaatcggcggaattaacaactcattgcctaacccacacttgagaaaggactc<br>caataatccagccttgcattttgaatgggaaatcattcatgcatttggcaaggacgcagaaggcgaacgtagttccgctataaacacgtcacctatttccgtggtggataaggaaagatt<br>cagcaaataccatgacaattactatcctggctggtatttggttgatgacactttagagagagcaggcaggatcaagaattaaATTGAATTGAATTGAAATCGATA<br>GATCAATTTTTTTCTTTTCTCTTTCCCCATCCTTTACGCTAAAATAATAGTTTATTTTATTTTTTGAATATTTTTT<br>ATTTATATACGTATATATAGACTATTATTTATCTTTTAATGATTATTAAGATTTTTATTAAAAAAAAAATTCGCTC<br>CTCTTTTAATGCCTTTATGCAGTTTTTTTTTCCCATTCGATATTTCTATGTTCGGGTTCAGCGTATTTTAAGTTTA<br>ATAACTCGAAAATTCTGCGTTCGTTAAAGCTTTCGAGAAGGATATTATTTCGAAATAAACCGTGTTGTGTAAG<br>CTTGAAGCCTTTTTGCGCTGCCAATATTCTTATCCATCTATTGTACTCTTTAGATCCAGTATAGTGTATTCTTCC<br>TG
Expression Modules for Lycopene Readout Optimization (pMJ006, pMJ009, pMJ012)	
pFUS1-CrtI-tACT1	Same as pFUS1-CrtI-tACT1 expression module in *Lycopene Readout Modules* above.
pTDH3-tHMG1-tCYC1	AGTTTATCATTATCAATACTCGCCATTTCAAAGAATACGTAAATAATTAATAGTAGTGATTTTCCTAACTTTAT<br>TTAGTCAAAAAATTAGCCTTTTAATTCTGCTGTAACCCGTACATGCCCAAAATAGGGGGCGGGTTACACAGAA<br>TATATAACATCGTAGGTGTCTGGGTGAACAGTTTATTCCTGGCATCCACTAAATATAATGGAGCCCGCTTTTTA<br>AGCTGGCATCCAGAAAAAAAAAAGAATCCCAGCACCAAAATATTGTTTTCTTCACCAACCATCAGTTCATAGGT<br>CCATTCTCTTAGCGCAACTACAGAGAACAGGGGCACAAACAGGCAAAAAACGGGCACAACCTCAATGGAGTG<br>ATGCAACCTGCCTGGAGTAAATGATGACACAAGGCAATTGACCCACGCATGTATCTATCTCATTTTCTTACAC<br>CTTCTATTACCTTCTGCTCTCTCTGATTTGGAAAAAGCTGAAAAAAAAAGGTTGAAACCAGTTCCCTGAAATTAT<br>TCCCCTACTTGACTAATAAGTATATAAAGACGGTAGGTATTGATTGTAATTCTGTAAATCTATTTCTTAAACTT<br>CTTAAATTCTACTTTTATAGTTAGTCTTTTTTTTAGTTTTAAAACACCAGAACTTAGTTTCGACGGATTCTAGAA<br>CTAGTatggaccaattggtgaaaactgaagtcaccaagaagtcttttactgctcctgtacaaaaggcttctacaccagttttaaccaataaaacagtcatttctggatcgaaagtcaa<br>aagtttatcatctgcgcaatcgagctcatcaggaccttcatcatctagtgaggaagatgattcccgcgatattgaaagcttggataagaaaatacgtcctttagaagaattagaagcattat<br>taagtagtggaaatacaaaacaattgaagaacaaagaggtcgctgccttggttattcacggtaagttacctttgtacgctttggagaaaaaattaggtgatactacgagagcggttgcgg<br>tacgtaggaaggctctttcaattttggcagaagctcctgtattagcatctgatcgtttaccatataaaaattatgactacgaccgcgtatttggcgcttgttgtgaaaatgttataggttacatg<br>cctttgcccgttggtgttataggccccttggttatcgatggtacatcttatcatataccaatggcaactacagagggttgtttggtagcttctgccatgcgtggctgtaaggcaatcaatgct<br>ggcggtggtgcaacaactgttttaactaaggatggtatgacaagaggcccagtagtccgtttcccaactttgaaaagatctggtgcctgtaagatatggttagactcagaagagggaca<br>aaacgcaattaaaaaagcttttaactctacatcaagatttgcacgtctgcaacatattcaaacttgtctagcaggagatttactcttcatgagatttagaacaactactggtgacgcaatggg<br>tatgaatatgatttctaaaggtgtcgaatactcattaaagcaaatggtagaagagtatggctgggaagatatggaggttgtctccgtttctggtaactactgtaccgacaaaaaaccagct<br>gccatcaactggatcgaaggtcgtggtaagagtgtcgtcgcagaagctactattcctggtgatgttgtcagaaaagtgttaaaaagtgatgtttccgcattggttgagttgaacattgcta<br>agaatttggttggatctgcaatggctgggtctgttggtggatttaacgcacatgcagctaatttagtgacagctgttttcttggcattaggacaagatcctgcacaaaatgttgaaagttcca<br>actgtataacattgatgaaagaagtggacggtgatttgagaatttccgtatccatgccatccatcgaagtaggtaccatcggtggtggtactgttctagaaccacaaggtgccatgttgg<br>acttattaggtgtaagaggcccgcatgctaccgctcctggtaccaacgcacgtcaattagcaagaatagttgcctgtgccgtcttggcaggtgaattatccttatgtgctgccctagcag<br>ccggccatttggttcaaagtcatatgacccacaacaggaaacctgctgaaccaacaaaacctaacaatttggacgccactgatataaatcgtttgaaagatgggtccgtcacctgcatt<br>aaatcctaaCTCGAGTCATGTAATTAGTTATGTCACGCTTACATTCACGCCCTCCCCCCACATCCGCTCTAACCGAA<br>AAGGAAGGAGTTAGACAACCTGAAGTCTAGGTCCCTATTTATTTTTTTATAGTTATGTTAGTATTAAGAACGTT<br>ATTTATATTTCAAATTTTTCTTTTTTTTCTGTACAGACGCGTGTACGCATGTAACATTATACTGAAAACCTTGCT<br>TGAGAAGGTTTTGGGACGCTCGAAGGCTTTAATTTGCGGCC

pTDH3-FAD1-tCYC1	AGTTTATCATTATCAATACTCGCCATTTCAAAGAATACGTAAATAATTAATAGTAGTGATTTTCCTAACTTTAT<br>TTAGTCAAAAAATTAGCCTTTTAATTCTGCTGTAACCCGTACATGCCCAAAATAGGGGGCGGGTTACACAGAA<br>TATATAACATCGTAGGTGTCTGGGTGAACAGTTTATTCCTGGCATCCACTAAATATAATGGAGCCCGCTTTTTA<br>AGCTGGCATCCAGAAAAAAAAAAGAATCCCAGCACCAAAATATTGTTTTCTTCACCAACCATCAGTTCATAGGT<br>CCATTCTCTTAGCGCAACTACAGAGAACAGGGGCACAAACAGGCAAAAAACGGGCACAACCTCAATGGAGTG<br>ATGCAACCTGCCTGGAGTAAATGATGACACAAGGCAATTGACCCACGCATGTATCTATCTCATTTTCTTACAC<br>CTTCTATTACCTTCTGCTCTCTCTGATTTGGAAAAAGCTGAAAAAAAAGGTTGAAACCAGTTCCCTGAAATTAT<br>TCCCCTACTTGACTAATAAGTATATAAAGACGGTAGGTATTGATTGTAATTCTGTAAATCTATTTCTTAAACTT<br>CTTAAATTCTACTTTTATAGTTAGTCTTTTTTTTAGTTTTAAAACACCAGAACTTAGTTTCGACGGATTCTAGAA<br>CTAGTAACatgcagttgagcaaggctgctgagatgtgttatgagataacaaactcttacttacacatagaccagaaatctcagataatagcaagtacacaagaagcgatacggt<br>tgacaagaaaatacttactaagtgaaatttttgtacgttggagtccactgaatggggaaatatcattctcgtacaacggaggaaaagattgccaggtattactactgttatatctgagttgct<br>tatgggaatatttcttcattaaggctcaaaattcccaattcgatttcgagtttcaaagcttccccatgcaaagacttccaactgttttcattgatcaagaagaaactttccctacattagagaatt<br>ttgtactggaaacctcagagcgatattgcctttccttatacgaatcacaaaggcaatctggtgcatcggtcaatatggcagacgcatttagagattttataaagatataccctgagaccga<br>agctatagtgataggtattagacacacagacccatttggtgaagcattaaagcctattcaaagaacagattctaactggcctgattttatgaggttgcaacctctcttacactgggacttaa<br>ccaatatatggagtttcttactgtattctaatgagccaatttgtggactatatggtaaaggtttcacatcaatcggcggaattaacaactcattgcctaacccacacttgagaaaggactcca<br>ataatccagccttgcattttgaatgggaaatcattcatgcatttggcaaggacgcagaaggcgaacgtagttccgctataaacacgtcacctatttccgtggtggataaggaaagattca<br>gcaaataccatgacaattactatcctggctggtatttggttgatgacactttagagagagcaggcaggatcaagaattaaCTCGAGTCATGTAATTAGTTATGTC<br>ACGCTTACATTCACGCCCTCCCCCCACATCCGCTCTAACCGAAAAGGAAGGAGTTAGACAACCTGAAGTCTAG<br>GTCCCTATTTATTTTTTTATAGTTATGTTAGTATTAAGAACGTTATTTATATTTCAAATTTTTCTTTTTTTTCTGT<br>ACAGACGCGTGTACGCATGTAACATTATACTGAAAACCTTGCTTGAGAAGGTTTTGGGACGCTCGAAGGCTTT<br>AATTTGCGGCC

table S5. Primers for cloning of fungal receptors and for genotyping of *C. albicans* isolates.
Gibson assembly was used for receptor cloning except where restriction sites are underlined.

Primers used for cloning fungal receptors from genomic DNA and pLPreB	
Sc.Ste2	MJ492 ACCAAGAACTTAGTTTCGACGGATACTAGTAAAATGTCTGATGCGGCTCCTTC<br>MJ493 ACGAAATTACTTTTTCAAAGCCGTCTCGAGCTATAAATTATTATTATCTTCAGTCCAGAA
Ca.Ste2	MJ440 acgtcaaggagaaaaaaccccggaaactagtaAAATGAATATCAATTCAACTTTCATACC<br>MJ362 gcaagtctcgagCTACACTCTTTTGATGGTGATTTG
Cg.Ste2	MJ498 ACCAAGAACTTAGTTTCGACGGATACTAGTAAAATGGAGATGGGCTACGATCC<br>MJ499 ACGAAATTACTTTTTCAAAGCCGTCTCGAGCTATTTGTCACACTGACTTTGTTG
Le.Ste2	MJ504 ACCAAGAACTTAGTTTCGACGGATACTAGTAAAATGGACGAAGCAATCAATGCAAAC<br>MJ505 ACGAAATTACTTTTTCAAAGCCGTCTCGAGCTATTTTTTCAACATAGTCACTTC
Pb.Ste2	MJ508 ACCAAGAACTTAGTTTCGACGGATACTAGTAAAATGGCACCCTCATTCGACC<br>MJ509 ACGAAATTACTTTTTCAAAGCCGTCTCGAGCTAGGCCTTTGTGCCAGCTTC
Zr.Ste2	MJ518 ACCAAGAACTTAGTTTCGACGGATACTAGTAAAATGAGTGAGATTAACAATTCTACCTAC<br>MJ519 ACGAAATTACTTTTTCAAAGCCGTCTCGAGCTATAATTTCTTTAGGATAATTTTTTTACT
Primers used for genotyping MTL loci of C. albicans	
MTLα	SB469 TGTAAACATCCTCAATTGTACCCGA<br>SB470 TTCGAGTACATTCTGGTCGCG
MTLa1	SB471 TTCGAGTACATTCTGGTCGCG<br>SB472 ATCAATTCCCTTTCTCTTCGATTAGG

table S6. DNA sequence for fungal receptor ORFs used in this study.

Description	Sequence
Yeast codon-optimized fungal receptor ORFs	
B. cinerea STE2 homologue	ATGGCTTCTAACTCTTCTAACTTCGACCCATTGACTCAATCTATCACTATCTTGATGGCTGACGGTATC<br>ACTACTGTTTCTTTCACTCCATTGGACATCGACTTCTTCTACTACTACAACGTTGCTTGTTGTATCAACT<br>ACGGTGCTCAAGCTGGTGCTTGTTTGTTGATGTTCTTCGTTGTTGTTGTTTTGACTAAGGCTGTTAAGA<br>GAAAGACTTTGTTGTTCGTTTTGAACGTTTTGTCTTTGATCTTCGGTTTCTTGAGAGCTATGTTGTACG<br>CTATCTACTTCTTGCAAGGTTTCAACGACTTCTACGCTGCTTTCACTTTCGACTTCTCTAGAGTTCCAA<br>GATCTTCTTACGCTTCTTCTGTTGCTGGTTCTGTTATCCCATTGTGTATGACTATCACTGTTAACATGTC<br>TTTGTACTTGCAAGCTTACACTGTTTGTAAGAACTTGGACGACATCAAGAGAATCATCTTGACTACTT<br>TGTCTGCTATCGTTGCTTTGTTGGCTATCGGTTTCAGATTCGCTGCTACTGTTGTTAACTCTGTTGCTAT<br>CTTGGCTACTTCTGCTTCTTCTGTTCCAATGCAATGGTTGGTTAAGGGTACTTTGGTTACTGAAACTAT<br>CTCTATCTGGTTCTTCTCTTTGATCTTCACTGGTAAGTTGGTTTGGACTTTGTACAACAGAAGAAGAAA<br>CGGTTGGAGACAATGGTCTGCTGTTAGAATCTTGGCTGCTATGGGTGGTTGTACTATGGTTATCCCAT<br>CTATCTTCGCTATCTTGGAATACGTTACTCCAGTTTCTTTCCCAGAAGCTGGTTCTATCGCTTTGACTTC<br>TGTTGCTTTGTTGTTGCCAATCTCTTCTTTGTGGGCTGGTATGGTTACTGACGAAGAAACTTCTGCTAT<br>CGACGTTTCTAACTTGACTGGTTCTAGAACTATGTTGGGTTCTCAATCTGGTAACTTCTCTAGAAAGAC<br>TCACGCTTCTGACATCACTGCTCAATCTTCTCACTTGGACTTCTCTTCTAGAAAGGGTTCTAACGCTAC<br>TATGATGAGAAAGGGTTCTAACGCTATGGACCAAGTTACTACTATCGACTGTGTTGTTGAAGACAACC<br>AAGCTAACAGAGGTTTGAGAGACTCTACTGAAATGGACTTGGAAGCTATGGGTGTTAGAGTTAACAA<br>GTCTTACGGTGTTCAAAAGGCTTAG
F. graminearum STE2 homologue	ATGTCTAAGGAAGTTTTCGACCCATTCACTCAAAACGTTACTTTCTTCGCTCCAGACGGTAAGACTGA<br>AATCTCTATCCCAGTTGCTGCTATCGACCAAGTTAGAAGAATGATGGTTAACACTACTATCAACTACG<br>CTACTCAATTGGGTGCTTGTTTGATCATGTTGGTTGTTTTGTTGGTTATGGTTCCAAAGGAAAAGTTCA<br>GAAGACCATTCATGATCTTGCAAATCACTTCTTTGGTTATCTCTTGTTGTAGAATGTTGTTGTTGTCTA<br>TCTTCCACTCTTCTCAATTCTTGGACTTCTACGTTTTCTGGGGTGACGACCACTCTAGAATCCCAAGAT<br>CTGCTTACGCTCCATCTGTTGCTGGTAACACTATGTCTTTGTGTTTGGTTATCTCTGTTGAAACTATGTT<br>GATGTCTCAAGCTTGGACTATGGTTAGATTGTGGCCAAACGTTTGGAAGTACATCATCGCTGGTGTTT<br>CTTTGATCGTTTCTATCATGGCTATCTCTGTTAGATTGGCTTACACTATCATCCAAAACAACGCTGTTT<br>TGAAGTTGGAACCAGCTTTCCACATGTTCTGGTTGATCAAGTGGACTGTTATCATGAACGTTGCTTCT<br>ATCTCTTGGTGGTGTGCTATCTTCAACATCAAGTTGGTTTGGCACTTGATCTCTAACAGAGGTATCTTG<br>CCATCTTACAAGACTTTCACTCCAATGGAAGTTTTGATCATGACTAACGGTATCTTGATGATCATCCC<br>AGTTATCTTCGCTTCTTTGGAATGGGCTCACTTCGTTAACTTCGAATCTGCTTCTTTGACTTTGACTTCT<br>GTTGCTGTTATCTTGCCATTGGGTACTTTGGCTGCTCAAAGAATCGCTTCTTCTGCTCCATCTTCTGCT<br>AACTCTACTGGTGCTTCTTCTGGTATCAGATACGGTGTTTCTGGTCCATCTTCTTTCACTGGTTTCAAG<br>GCTCCATCTTTCTCTACTGGTACTACTGACAGACCACACGTTTCTATCTACGCTAGATGTGAAGCTGGT<br>ACTTCTTCTAGAGAACACATCAACCCACAAGGTGTTGAATTGGCTAAGTTGGACCCAGAAACTGACC<br>ACCACGTTAGAGTTGACAGAGCTTTCTTGCAAAGAGAAGAAAGAATCAGAGCTCCATTGTAG
M. oryzea STE2 homologue	ATGGACCAAACTTTGTCTGCTACTGGTACTGCTACTTCTCCACCAGGTCCAGCTTTGACTGTTGACCCA<br>AGATTCCAAACTATCACTATGTTGACTCCAGCTTTGATGGGTCAAGGTTTCGAAGAAGTTCAAACTAC<br>TCCAGCTGAAATCAACGACGTTTACTTCTTGGCTTTCAACACTGCTATCGGTTACTCTACTCAAATCGG<br>TGCTTGTTTCATCATGTTGTTGGTTTTGTTGACTATGACTGCTAAGGCTAGATTCGCTAGAATCCCAAC<br>TATCATCAACACTGCTGCTTTGGTTGTTTCTATCATCAGATGTACTTTGTTGGTTATCTTCTTCACTTCT<br>ACTATGATGGAATTCTACACTATCTTCTCTGACGACTTCTCTTTCGTTCACCCAAACGACATCAGAAG<br>ATCTGTTGCTGCTACTGTTTTCGCTCCATTGCAATTGGCTTTGGTTGAAGCTGCTTTGATGGTTCAAGC<br>TTGGGCTATGGTTGAATTGTGGCCAAGAGCTTGGAAGGTTTCTGGTATCGCTTTCTCTTTGATCTTGGC<br>TACTGTTACTGTTGCTTTCAAGTGTGCTTCTGCTGCTGTTACTGTTAAGTCTGCTTTGGAACCATTGGA<br>CCCAAGACCATACTTGTGGATCAGACAAACTGACTTGGCTTTCACTACTGCTATGGTTACTTGGTTCT<br>GTTTCTTGTTCAACGTTAGATTGATCATGCACATGTGGCAAAACAGATCTATCTTGCCAACTGTTAAG<br>GGTTTGTCTCCAATGGAAGTTTTGGTTATGGCTAACGGTTTGTTGATGGTTTTCCCAGTTTTGTTCGCT<br>GGTTTGTACTACGGTAACTTCGGTCAATTCGAATCTGCTTCTTTGACTATCACTTCTGTTGTTTTGGTTT<br>TGCCATTGGGTACTTTGGTTGCTCAAAGATTGGCTGTTAACAACACTGTTGCTGGTTCTTCTGCTAACA<br>CTGACATGGACGACAAGTTGGCTTTCTTGGGTAACGCTACTACTGTTACTTCTTCTGCTGCTGGTTTCG<br>CTGGTTCTTCTGCTTCTGCTACTAGATCTAGATTGGCTTCTCCAAGACAAAACTCTCAATTGTCTACTT<br>CTGTTTCTGCTGGTAAGCCAAGAGCTGACCCAATCGACTTGGAATTGCAAAGAATCGACGACGAAGA<br>CGACGACTTCTCTAGATCTGGTTCTGCTGGTGGTGTTAGAGTTGAAAGATCTATCGAAAGAAGAGAA<br>GAAAGATTGTAG
Z. bailii STE2 homologue	ATGTCTGGTTTGGCTAACAACACCTCTTACAACCCATTGGAATCTTTCATTATTTTCACTTCTGTTTAC<br>GGTGGTGATACCATGGTTAAGTTCGAAGACTTGCAATTAGTCTTCACCAAGCGTATTACTGAAGGTAT<br>TTTGTTCGGTGTCAAGGTTGGTGCCGCTTCTTTGACTATGATTGTTATGTGGATGATTTCCAGAAGAAG<br>AACCTCCCCAATCTTCATCATGAACCAATTGTCTTTGGTTTTCACCATCTTGCACGCTTCTTTTTACTTT<br>AAGTACTTATTGGACGGTTTCGGTTCTATTGTCTACACTTTGACCTTGTTCCCACAATTAATTACTTCC<br>TCTGACTTGCACGTTTTCGCTACTGCTAACGTTGTTGAAGTCTTATTGGTTTCTTCCATCGAAGCCTCTT<br>TGGTTTTCCAAGTCAACGTCATGTTCGCTGGTTCTAACCACAGAAAGTTCGCTTGGTTGTTGGTCGGTT<br>TCTCTTTGGGTTTGGCTTTGGCCACTGTCGCTTTGTACTTCGTTACTGCTGTCAAGATGATCGCTTCCG<br>CTTACGCTTCTCAACCACCAACTAACCCAATCTACTTCAACGTTTCCTTGTTCTTGTTGGCTGCCTCCG<br>TTTTCTTGATGACTTTAATGTTGACCGTCAAGTTGATCTTGGCTATCAGATCCAGAAGATTCTTGGGTT<br>TGAAGCAATTCGACTCTTTCCACATTTTGTTGATTATGTCTTGTCAAACTTTGATCGCTCCATCTGTTTT<br>GTACATCTTGGGTTTTATTTTGGATCACAGAAAGGGTAACGACTACTTGATTACCGTCGCTCAATTGTT<br>GGTCGTTTTGTCTTTGCCATTGTCCTCCATGTGGGCCACTACTGCTAACGATGCTTCCTCCGGTACTTC<br>TATGTCTTCCAAGGAATCCGTCTACGGTTCTGATTCCTTATACTCTAAGTCTAAGTGTTCCCAATTCAC

	CAGAACCTTCATGAACAGATTCTCTACTAAGCCAACTAAGAACGACGAAATTTCTGATTCCGCTTTCG<br>TCGCTGTTGATTCCTTGGAAAAGAACGCTCCACAAGGTATCTCTGAACACGTTTGTGAATTCCCACAA<br>TCTGACTTATCTGATCAAGCTACTTCCATCTCCTCCAGAAAAAAGGAAGCTGTTGTTTACGCTTCCACT<br>GTTGATGAAGATAAGGGTTCTTTCTCCTCTGACATCAACGGTTACACTGTTACCAACATGCCATTGGC<br>TTCCGCTGCTTCTGCTAACTGTGAAAACTCCCCATGTCACGTTCCAAGACCATACGAAGAAAACGAAG<br>GTGTCGTCGAAACCAGAAAAATTATTTTGAAGAAGAACGTCAAATGGTAG
H. capsulatum<br>STE2 homologue	ATGTCTTCCTCTTTCGACCCATTCGACCAAAACGTTGTCTTCCACAAGGCTGATGGTACTCCATTCAAC<br>GTTTCCATTCACGACTTGGACGAATTCGTCCAATACGGTATTAGAGTCTGTATCAACTACGCTGCTCA<br>ATTGGGTGCCACTGTCATTGCTATTGTCATGTTGGCTTTGTTGACTCAATCCGATAAGAGAAGAACCC<br>CAGTCTTCTTCTTGAACACTTCTGCTTTGACTATGAACTTCGCTAGATTGTTGTGTATGACTATTTACTT<br>CACCACTGGTTTCAACTCTACCTACGCTTTCTTCTCTTTGGACTACTCCAGAGTCCCAGGTTCTGCCTA<br>CGCTGATTCTATCTTGGGTATCGCTTTCGCTACTATTTTGGTCATTTGTATGGAAATGTCTTTGGTTATT<br>CAAACTCAAGTTGTTTGTGCCACCTTGTCTGAAATCCAAAGAAGATTGTTGTTGGTCGTCTCCATTTTG<br>ATCGCTTTGTTGGCTATTGGTTTCAGAATGGGTTTGATGGTTGAAAACTGTATCGCTATCATGAACGC<br>CTCTAACTTCAGACCATTCATCTGGTTGCAATCCGCTTCTAACATTGCTATTACCATCTCTACTTGTTTC<br>TTCTCTGCTGTTTTCGTCACCAAGTTGGGTTACGCTTTGGTTACCAGAAGAAGATTGGGTATGACTAG<br>ATTCGGTGCTATGCAAGTCATGTTCATTATGTCCTTCCAAACTATGGTCATCCCAGCTATTTTCTCCAT<br>TATCCAATACCCAATCCCATTGTACGAAATGAACTCTAACGTCTTCACTTTGGTCGCTATTTTCTTGCC<br>ATTGTCTTCCTTGTGGGCCGCTGCTGCTACTAAGCACTCCTTCGAAACTTTGACCTCTGGTCCACACCA<br>ATACTTGTGGTCCTCTGAAAGATCCAACTCTACCTCTTCCGCTACCGGTCACCAAGGTTCTTTGTGTCA<br>AAACCAATCTACTATCAGATCTGGTGGTTCTGTTGCTACTTCCTTGTCCCCAGACCAATTGGACAGATT<br>GTACACTGGTTTGGACTTCGACGCCTGTGCCAAGGCTTAG
C. glabrata<br>STE2 homologue	ATGGAAATGGGTTACGACCCAAGAATGTACAACCCAAGAAACGAATACTTGAACTTCACTTCTGTTT<br>ACGACGTTAACGACACTATCAGATTCTCTACTTTGGACGCTATCGTTAAGGGTTTGTTGAGAATCGCT<br>ATCGTTCACGGTGTTAGATTGGGTGCTATCTTCATGACTTTGATCATCATGTTCATCTCTTCTAACACT<br>TGGAAGAAGCCAATCTTCATCATCAACATGGTTTCTTTGATGTTGGTTATGATCCACTCTGCTTTGTCT<br>TTCCACTACTTGTTGTCTAACTACTCTTCTATCTCTTACATCTTGACTGGTTTCCCACAATTGATCACTT<br>CTAACAACAAGAGAATCCAAGACGCTGCTTCTATCGTTCAAGTTTTGTTGGTTGCTGCTATCGAAGCT<br>TCTTTGGTTTTCCAAATCCACGTTATGTTCACTATCGAAAACATCAAGTTGATCAGAGAAATCGTTTTG<br>TCTATCTCTATCGCTATGGGTTTGGCTACTGTTGCTACTTACTTGGCTGCTGCTATCAAGTTGATCAGA<br>GGTTTGCACGACGAAGTTATGCCACAAACTCACTTGATCTTCAACTTGTCTATCATCTTATTGGCTTCT<br>TCTATCAACTTCATGACTTTCATCTTAGTTATCAAGTTGTTCTTCGCTATCAGATCTAGAAGATACTTA<br>GGTTTGAGACAATTCGACGCTTTCCACATCTTGTTGATCATGTTCTGTCAATCTTTGTTGATCCCATCT<br>GTTTTGTACATCATCGTTTACGCTGTTGACTCTAGATCTAACCAAGACTACTTGATCCCAATCGCTAAC<br>TTGTTCGTTGTTTTGTCTTTGCCATTGTCTTCTATCTGGGCTAACACTTCTAACAACTCTTCTAGATCTC<br>CAAAGTACTGGAAGAACTCTCAAACTAACAAGTCTAACGGTTCTTTCGTTTCTTCTATCTCTGTTAACT<br>CTGACTCTCAAAACCCATTGTACAAGAAGATCGTTAGATTCACTTCTAAGGGTGACACTACTAGATCT<br>ATCGTTTCTGACTCTACTTTGGCTGAAGTTGGTAAGTACTCTATGCAAGACGTTTCTAACTCTAACTTC<br>GAATGTAGAGACTTGGACTTCGAAAAGGTTAAGCACACTTGTGAAAACTTCGGTAGAATCTCTGAAA<br>CTTACTCTGAATTGTCTACTTTGGACACTACTGCTTTGAACGAAACTAGATTGTTCTGGAAGCAACAA<br>TCTCAATGTGACAAGTAG
Fungal receptor ORFs from genomic DNA (stop codons changed to TAG)	
Wildtype<br>*S. cerevisiae* STE2	ATGTCTGATGCGGCTCCTTCATTGAGCAATCTATTTTATGATCCAACGTATAATCCTGGTCAAAGCAC<br>CATTAACTACACTTCCATATATGGGAATGGATCTACCATCACTTTCGATGAGTTGCAAGGTTTAGTTA<br>ACAGTACTGTTACTCAGGCCATTATGTTTGGTGTCAGATGTGGTGCAGCTGCTTTGACTTTGATTGTCA<br>TGTGGATGACATCGAGAAGCAGAAAAACGCCGATTTTCATTATCAACCAAGTTTCATTGTTTTTAATC<br>ATTTTGCATTCTGCACTCTATTTTAAATATTTACTGTCTAATTACTCTTCAGTGACTTACGCTCTCACCG<br>GATTTCCTCAGTTCATCAGTAGAGGTGACGTTCATGTTTATGGTGCTACAAATATAATTCAAGTCCTTC<br>TTGTGGCTTCTATTGAGACTTCACTGGTGTTTCAGATAAAAGTTATTTTCACAGGCGACAACTTCAAA<br>AGGATAGGTTTGATGCTGACGTCGATATCTTTCACTTTAGGGATTGCTACAGTTACCATGTATTTTGTA<br>AGCGCTGTTAAAGGTATGATTGTGACTTATAATGATGTTAGTGCCACCCAAGATAAATACTTCAATGC<br>ATCCACAATTTTACTTGCATCCTCAATAAACTTTATGTCATTTGTCCTGGTAGTTAAATTGATTTTAGC<br>TATTAGATCAAGAAGATTCCTTGGTCTCAAGCAGTTCGATAGTTTCCATATTTTACTCATAATGTCATG<br>TCAATCTTTGTTGGTTCCATCGATAATATTCATCCTCGCATACAGTTTGAAACCAAACCAGGGAACAG<br>ATGTCTTGACTACTGTTGCAACATTACTTGCTGTATTGTCTTTACCATTATCATCAATGTGGGCCACGG<br>CTGCTAATAATGCATCCAAAACAAACACAATTACTTCAGACTTTACAACATCCACAGATAGGTTTTAT<br>CCAGGCACGCTGTCTAGCTTTCAAACTGATAGTATCAACAACGATGCTAAAAGCAGTCTCAGAAGTA<br>GATTATATGACCTATATCCTAGAAGGAAGGAAACAACATCGGATAAACATTCGGAAAGAACTTTTGT<br>TTCTGAGACTGCAGATGATATAGAGAAAAATCAGTTTTATCAGTTGCCCACACCTACGAGTTCAAAAA<br>ATACTAGGATAGGACCGTTTGCTGATGCAAGTTACAAAGAGGGAGAAGTTGAACCCGTCGACATGTA<br>CACTCCCGATACGGCAGCTGATGAGGAAGCCAGAAAGTTCTGGACTGAAGATAATAATAATTTATAG
C. glabrata<br>STE2 homologue	ATGGAGATGGGCTACGATCCAAGAATGTATAATCCAAGAAATGAATACTTGAATTTCACGTCGGTAT<br>ATGATGTAAATGACACAATCAGATTTTCGACTCTGGACGCCATTGTAAAAGGATTGCTTAGAATTGCC<br>ATTGTTCATGGAGTTAGATTGGGAGCAATATTCATGACGTTAATAATAATGTTTATCTCATCAAATAC<br>ATGGAAAAAACCCATATTTATAATTAACATGGTGTCGTTGATGTTAGTTATGATTCATTCCGCACTTA<br>GCTTCCATTACCTTTTATCGAATTATTCTTCAATTTCTTATATACTGACAGGGTTTCCTCAGTTGATTAC<br>AAGCAATAATAAACGAATTCAAGATGCAGCGAGTATAGTCCAAGTTTTATTGGTTGCTGCGATAGAA<br>GCATCATTGGTATTTCAGATTCATGTTATGTTTACGATTGAAAACATTAAGCTTATTAGAGAAATAGT<br>ACTCTCTATATCGATAGCAATGGGATTGGCAACAGTGGCTACATATCTTGCTGCAGCAATAAAGCTGA<br>TAAGAGGACTGCATGATGAGGTAATGCCACAAACACATCTTATTTTCAATTTATCTATAATATTGCTT<br>GCATCCTCCATAAATTTTATGACATTTATATTGGTCATTAAACTTTTCTTCGCTATTAGATCTAGAAGA<br>TATCTCGGTCTTCGTCAATTCGATGCTTTTCATATTTTATTAATCATGTTCTGCCAGTCATTATTGATAC<br>CCTCAGTATTATATATTATAGTTTACGCGGTTGATAGCAGATCTAATCAGGATTATCTGATTCCAATTG<br>CCAATTTATTTGTTGTTTTATCTTTGCCATTATCCTCTATCTGGGCTAACACATCAAATAACTCATCCA

	GATCTCCAAAATATTGGAAAAACTCTCAAACGAATAAGAGCAATGGGTCTTTTGTCTCTTCAATATCT<br>GTCAATAGTGACTCACAAAACCCTTTGTACAAAAAGATTGTACGTTTTACATCAAAAGGCGACACTAC<br>CCGTAGTATTGTAAGTGATTCAACATTAGCAGAGGTGGGAAAATACTCTATGCAAGACGTTAGCAAT<br>TCAAACTTTGAATGTCGAGACCTTGATTTTGAGAAGGTAAAACATACTTGCGAAAATTTTGGCAGAAT<br>ATCTGAAACATATAGTGAGTTAAGTACTTTAGATACCACTGCCCTCAATGAGACTCGGTTGTTTTGGA<br>AACAACAAAGTCAGTGTGACAAATAG
C. albicans<br>STE2 homologue	ATGAATATCAATTCAACTTTCATACCTGATAAACCAGGCGATATAATTATTAGTTATTCAATTCCAGG<br>ATTAGATCAACCAATTCAAATTCCTTTCCATTCATTAGATTCATTTCAAACCGATCAAGCTAAAATAG<br>CTTTAGTCATGGGGATAACTATTGGGAGTTGTTCAATGACATTAATTTTTTTGATTTCTATAATGTATA<br>AAACTAATAAATTAACAAATTTAAAATTAAAATTAAAATTAAAATATATCTTGCAATGGATAAATCA<br>AAAAATCTTCACCAAAAAAAGGAATGACAACAAACAACAACAACAACAACAACAACAACAAATTGA<br>ATCATCATCATATAACAATACTACTACTACGCTGGGGGGTTATAAATTATTTTTATTTTATCTTAATTC<br>ATTGATTTTATTAATTGGTATTATTCGATCAGGTTGTTATTTAAATTATAATTTAGGTCCATTAAATTC<br>ACTTAGTTTTGTATTTACTGGTTGGTATGATGGATCATCATTTATATCATCCGATGTAACTAATGGATT<br>TAAATGTATTTTATATGCTTTAGTGGAAATTTCATTAGGTTTCCAAGTTTATGTGATGTTCAAAACTTC<br>AAATTTAAAAATTTGGGGGATAATGGCATCATTATTATCAATTGGTTTAGGATTGATTGTTGTTGCCTT<br>TCAAATCAATTTAACAATTTTATCTCATATTCGATTTTCCCGGGCTATATCAACTAACAGAAGTGAAG<br>AAGAATCATCATCATCATTATCATCTGATTCGGTTGGGTATGTGATTAATTCAATATGGATGGATTTA<br>CCAACAATATTATTTTCCATTAGTATTAATATAATGACAATATTATTGATTGGTAAACTTATAATTGCT<br>ATTAGAACAAGACGTTATTTAGGATTGAAACAATTTGATAGTTTCCATATTTTATTAATTGGTTTCAGT<br>CAAACATTAATTATTCCTTCAATTATTTTGGTGGTTCATTATTTTTATTTATCACAAAATAAAGATTCTT<br>TATTACAACAAATTAGTCTTTTATTGATTATTTTAATGTTACCATTAAGTTCTTTATGGGCTCAAACTG<br>CTAATAATACTCATAATATTAATTCATCTCCAAGTTTATCATTCATATCTCGTCATCATCTGTCTGATA<br>GTAGTCGTAGTGGTGGTTCCAATACAATTGTTAGTAATGGTGGTAGTAATGGTGGTGGTGGTGGTGGT<br>GGGAATTTCCCTGTTTCAGGTATTGATGCACAATTACCACCTGATATTGAAAAAATCTTACATGAAGA<br>TAATAATTATAAATTACTTAATAGTAATAATGAAAGTGTAAATGATGGAGATATTATCATTAATGATG<br>AAGGTATGATTACTAAACAAATCACCATCAAAAGAGTGTAG
L. elongisporus<br>STE2 homologue	ATGGACGAAGCAATCAATGCAAACCTTGTTTCTGGAGATATTATAGTCTCTTTTAACATTCCTGGTTTG<br>CCAGAACCGGTACAAGTGCCATTCAGCGAATTTGATTCGTTTCATAAAGACCAGCTCATTGGAGTCAT<br>CATTCTTGGAGTCACTATTGGAGCATGCTCGCTTTTGTTGATATTGCTACTTGGAATGTTATACAAGAG<br>CCGTGAAAAGTATTGGAAATCACTATTATTTATGCTCAATGTATGCATCTTGGCTGCCACAATCTTAA<br>GGAGCGGTTGCTTCTTAGACTATTATCTAAGTGATTTGGCCAGTATCAGTTATACATTTACTGGAGTAT<br>ACAATGGTACCAGCTTTGCTAGCTCTGACGCGGCAAATGTGTTCAAGACTATTATGTTTGCCTTGATT<br>GAAACTTCGTTAACCTTTCAAGTGTATGTCATGTTTCAAGGGACCACTTGGAAAAATTGGGGCCATGC<br>TGTCACTGCATTATCGGGTCTCTTGTCTGTTGCCTCAGTGGCGTTCCAGATCTACACCACGATTTTATC<br>CCACAATAATTTCAATGCTACAATCTCGGGAACCGGTACATTAACTTCAGGTGTTTGGATGGACTTAC<br>CAACACTCTTGTTTGCCGCAAGTATCAATTTTATGACCATTTTGTTGTTATTTAAGTTGGGAATGGCCA<br>TTAGACAAAGAAGGTATTTAGGTTTAAAACAGTTTGATGGGTTCCATATCTTATTCATCATGTTTACCC<br>AAACATTGTTCATACCCTCGATTTTGCTTGTGATCCACTACTTTTACCAGGCAATGTCTGGACCATTCA<br>TCATCAACATGGCGTTGTTCTTGGTGGTGGCATTCTTGCCATTGAGTTCATTATGGGCACAAACTGCA<br>AACACTACTAAAAAGATTGAATCTTCGCCAAGTATGAGCTTTATTACTAGACGAAAATCAGAGGATG<br>AGTCACCACTGGCTGCTAACGACGAGGATAGGTTACGAAAATTCACCACAACTTTGGATTTGTCGGG<br>CAACAAGAACAATACAACAAACAATAATAACAATAGCAACAACATTAACAACAATATGAGCAACAT<br>CAACTACCCTTCTACAGGACTGGGAGAAGACGATAAATCCTTTATATTTGAGATGGAACCCAGTCGG<br>GAAAGAGCTGCAATAGAAGAGATTGATCTTGGAGCAAGGATCGATACCGGTTTGCCCAGAGATTTAG<br>AGAAATTTCTAGTTGATGGGTTTGACGATAGTGATGACGGAGAAGGAATGATAGCCAGAGAAGTGAC<br>TATGTTGAAAAAATAG
P. brasiliensis (lutzii)<br>STE2 homologue	ATGGCACCCTCATTCGACCCCTTCAACCAAAGCGTGGTCTTCCACAAGGCCGACGGAACTCCATTCAA<br>CGTCTCAATCCATGAACTAGACGACTTCGTGCAGTACAACACCAAAGTCTGCATCAACTACTCTTCCC<br>AGCTCGGAGCATCTGTCATTGCAGGACTCATGCTTGCCATGCTGACACACTCAGAAAAGCGTCGTCTG<br>CCAGTTTTCTTCCTAAACACATTCGCACTGGCCATGAACTTTGCCCGCCTGCTCTGCATGACCATCTAC<br>TTCACCACGGGCTTCAACAAGTCCTATGCCTACTTTGGTCAGGATTACTCCCAGGTGCCTGGGAGCGC<br>CTACGCAGCCTCTGTCTTGGGCGTTGTCTTCACCACTCTCCTGGTAATCAGCATGGAAATGTCCCTCCT<br>GATCCAAACAAGGGTTGTCTGCACGACCCTTCCGGATATCCAACGTTATCTACTCATGGCAGTTTCCT<br>CCGCGATTTCCCTGATGGCCATCGGGTTCCGCCTTGGCTTAATGGTTGAGAACTGCATTGCCATTGTG<br>CAGGCGTCGAATTTCGCCCCTTTTATCTGGCTTCAAAGCGCCTCGAACATCACCATTACGATCAGCAC<br>ATGTTTCTTCAGTGCCGTCTTTGTTACGAAATTGGCATATGCACTCGTCACTCGTATACGACTAGGCTT<br>GACGAGGTTTGGTGCTATGCAGGTTATGTTCATCATGTCCTGCCAGACTATGGTGATTCCAGCCATCT<br>TCTCAATTCTCCAATACCCACTCCCCAAGTACGAAATGAACTCCAACCTCTTTACGCTGGTGGCCATTT<br>TCCTCCCTCTTTCCTCGCTATGGGCTTCAGTTGCTACGAGATCCAGTTTCGAGACGTCTTCTTCCGGCC<br>GCCATCAGTATCTTTGGCCAAGCGAACAGAGCAATAACGTCACCAATTCGGAAATTAAGTATCAGGT<br>CAGCTTCTCTCAGAACCACACTACGTTGCGGTCTGGAGGGTCTGTGGCCACGACACTCTCCCCGGACC<br>GGCTCGACCCGGTTTATTGTGAAGTTGAAGCTGGCACAAAGGCCTAG
Z. rouxii<br>STE2 homologue	ATGAGTGAGATTAACAATTCTACCTACAATCCAATGAATGCATATGTAACGTTTACATCAATATATGG<br>TGATGATACTATGGTACGTTTCAAAGATGTGGAATTGGTAGTTAACAAAAGGGTTACAGAAGCCATT<br>ATGTTCGGCGTCAAAGTTGGTGCAGCTTCGTTGACACTCATCATCATGTGGATGATCTCTAAGAAAAG<br>AACAACACCGATATTTATCATAAATCAGTCTTCGCTTGTATTTACCATAATACATGCTTCGCTTTATTT<br>TGGGTACCTTTTGTCAGGATTTGGTAGTATAGTTTACAATATGACATCGTTCCCGCAGTTAATAAGCTC<br>CAATGACGTTCGTGTGTACGCAGCTACAAATATTTTTGAGGTCCTGTTGGTAGCATCTATCGAAATCT<br>CTCTGGTTTTTCAGGTCAAAGTTATGTTTGCCAACAATAATGGTCGAAGATGGACTTGGTGTTTGATG<br>GTAGTTTCCATAGGGATGGCACTAGCTACTGTAGGACTTTATTTTGCCACTGCCGTTGAGTTGATCAG<br>AGCTGCTTACAGCAATGATACTGTTAGCCGCCATGTTTTTTACAATGTTTCTCTGATCTTACTAGCGTC<br>ATCTGTCAATCTAATGACACTAATGCTAGTGGTAAAATTAGTATTAGCGATCAGATCAAGAAGATTTT<br>TGGGGTTAAAACAGTTTGACAGTTTCCACATATTACTTATAATGTCTTGCCAGACTCTAATAGCACCTT

	CCATTCTATTCATTTTGGGTTGGACCTTAGACCCTCATACTGGTAATGAGGTTTTAATTACAGTTGGTC<br>AATTGCTAATAGTACTGTCATTACCGCTGTCATCTATGTGGGCTACAACCGCTAACAATACCAGTTCA<br>TCTAGTAGTTCGGTGTCCTGTAATGACAGCTCTTTTGGTAATGACAATCTCTGTTCCAAGAGTTCGCAA<br>TTTAGAAGAACTTTTATGAATAGATTCCGTCCCAAGTCGGTTAATGGTGACGGTAATTCTGAAAATAC<br>CTTTGTTACAATTGATGATTTGGAAAAAAGCGTTTTTCAAGAATTATCAACACCTGTTAGCGGAGAAT<br>CAAAGATAGATCATGATCATGCAAGTAGTATTTCATGTCAAAAGACATGTAATCATGTTCATGCTTCG<br>ACAGTGAATTCAGATAAGGGATCTTGGTCCTCTGATGGTAGTTGTGGCAGTTCTCCGTTAAGAAAGAC<br>TTCCACCGTTAATTCTGAAGATTTACCTCCACATATATTGAGCGCCTACGATGACGATCGAGGTATAG<br>TAGAAAGTAAAAAAATTATCCTAAAGAAATTATAG

Supplementary File Captions

movie S1. Yeast dipstick assay with plastic holder. Dipstick with plastic holder dipped into YPD media containing either no ligand (water) or 1 µM of the synthetic fungal pathogen peptides from either *C. albicans* or *P. brasiliensis*.

movie S2. Yeast dipstick assay in soil. Dipstick inserted into soil preconditioned with either no ligand (water) or 2 nmol of synthetic fungal pathogen peptides from either *C. albicans* or *P. brasiliensis.* 2 mL of YPD media was added to the soil to initiate the assay giving an expected peptide concentration of 1 µM.

movie S3. Yeast dipstick assay in urine. Dipstick dipped into 50% human urine supplemented with YPD media and either no ligand (water) or 1 µM of the synthetic fungal pathogen peptides from either *C. albicans* or *P. brasiliensis*.

movie S4. Yeast dipstick assay in serum. Dipstick dipped into 50% human serum supplemented with YPD media and either no ligand (water) or 1 µM of the synthetic fungal pathogen peptides from either *C. albicans* or *P. brasiliensis*.

movie S5. Yeast dipstick assay in blood. Dipstick dipped into 2% human blood supplemented with YPD media and either no ligand (water) or 1 µM of the synthetic fungal pathogen peptides from either *C. albicans* or *P. brasiliensis*.
